# Supplementary material for: Inhibition of delta-secretase improves cognitive functions in mouse models of Alzheimer's disease
Source: Nat Commun. 2017 Mar 27;8:14740. doi: 10.1038/ncomms14740 (PMC5378956; doi:10.1038/ncomms14740)
Supplement: Supplementary Information — Supplementary Figures and Supplementary Tables [file ncomms14740-s1.pdf]

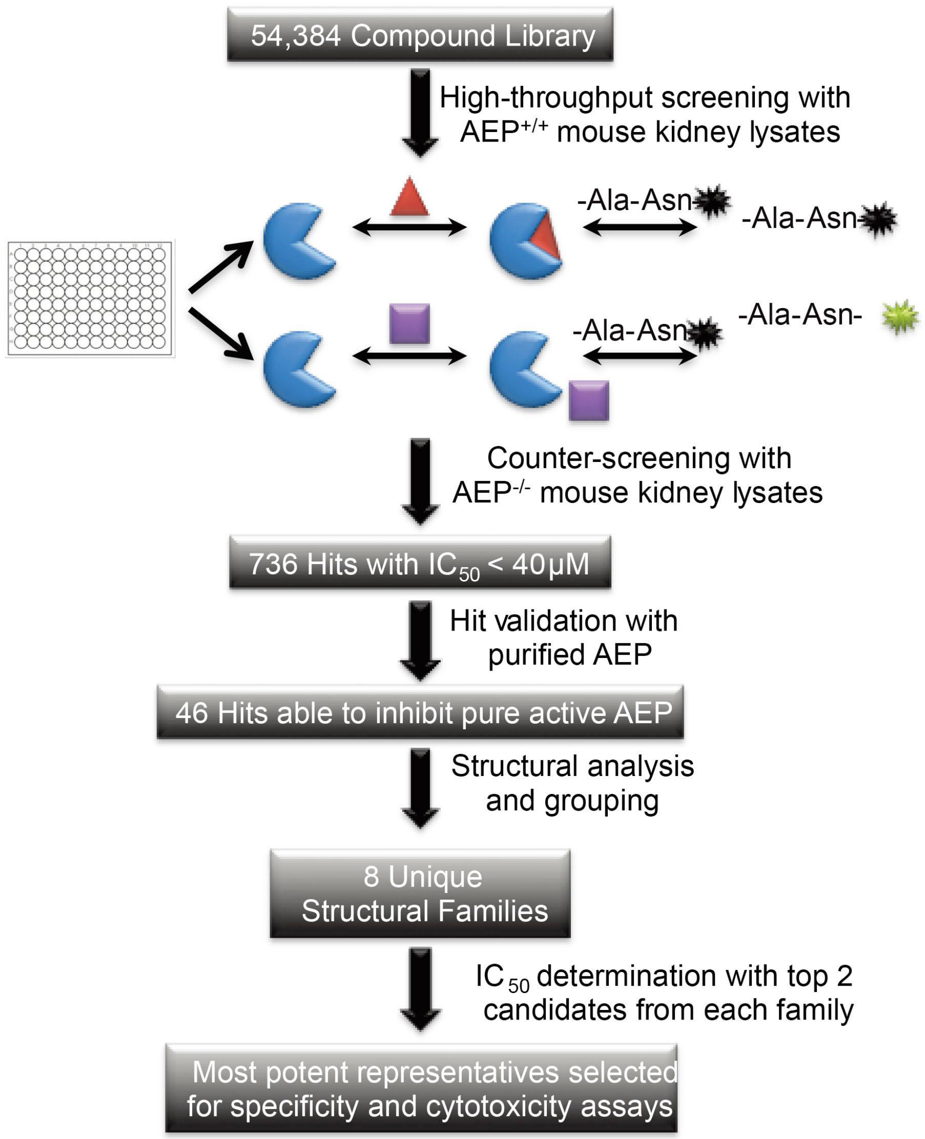

Supplementary Figure 1. The high-throughput screening scheme. An Asinex library of 54,384 compounds was screened with mouse kidney lysates, then counter-screened with δ-secretase knock-out lysates to yield 736 hits with IC_{50} values less than or equal to 40 μM. The hits were validated further with purified human δ-secretase, and promising compounds were categorized into 8 groups based on structural similarities. Two of the most potent compounds from each group were tested and the cytotoxicity and specificity were determined.

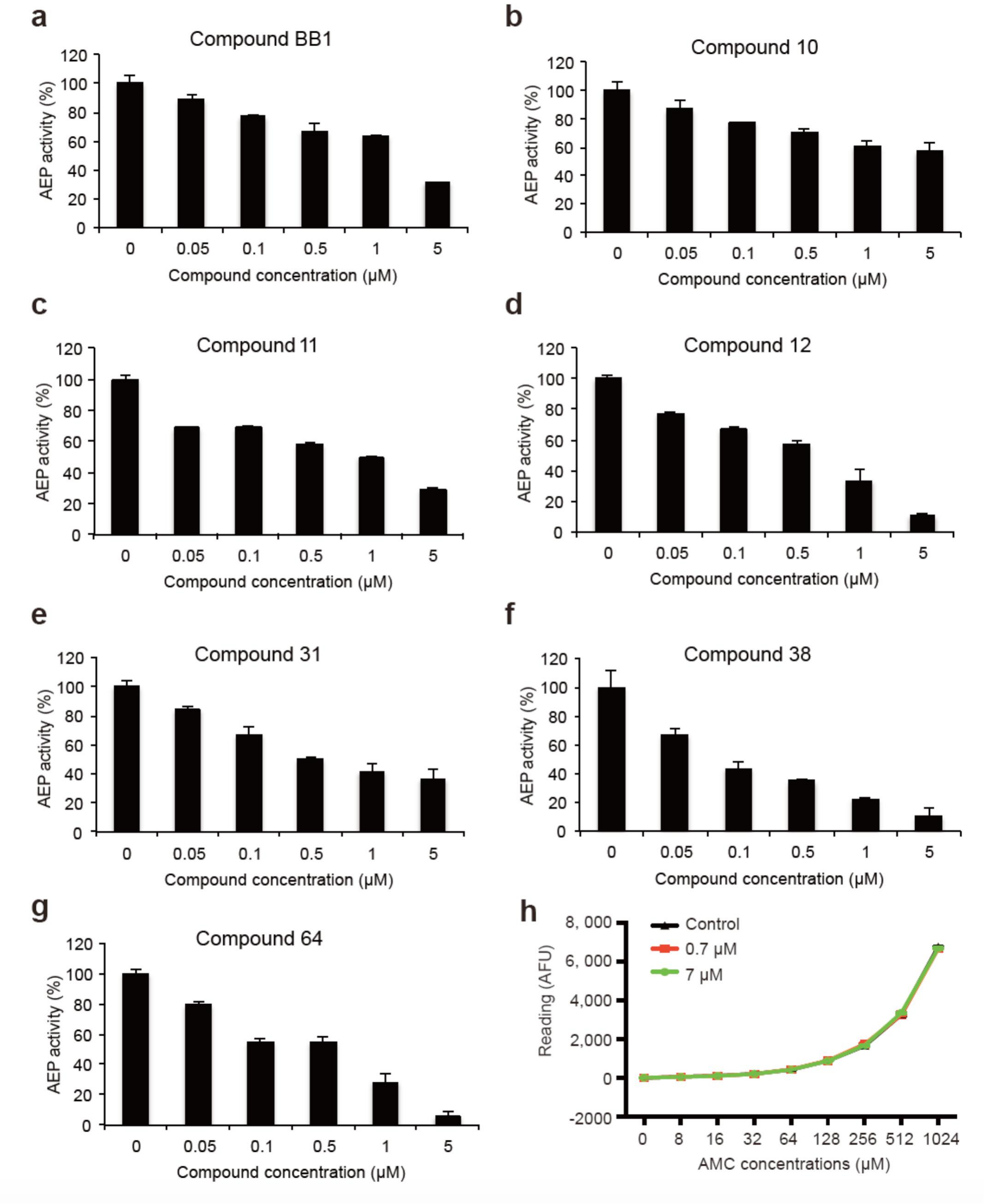

Supplementary Figure 2. Determination of IC50 values in intact Pala cells.

(**a-g**)Pala cells were incubated with different concentrations of compound for 2 h. Then the cells were washed, harvested, and the residual enzymatic activity was determined by δ-secretase activity assay. Data represent mean ± s.d. of three independent experiments.

(**h**) Compound 11 does not affect the fluorescent reading of AMC. 0.7 or 7 μM of compound 11 was incubated with different concentrations of AMC and read at the same condition as in AEP activity assay. The fluorescent reading was not affected by compound 11.

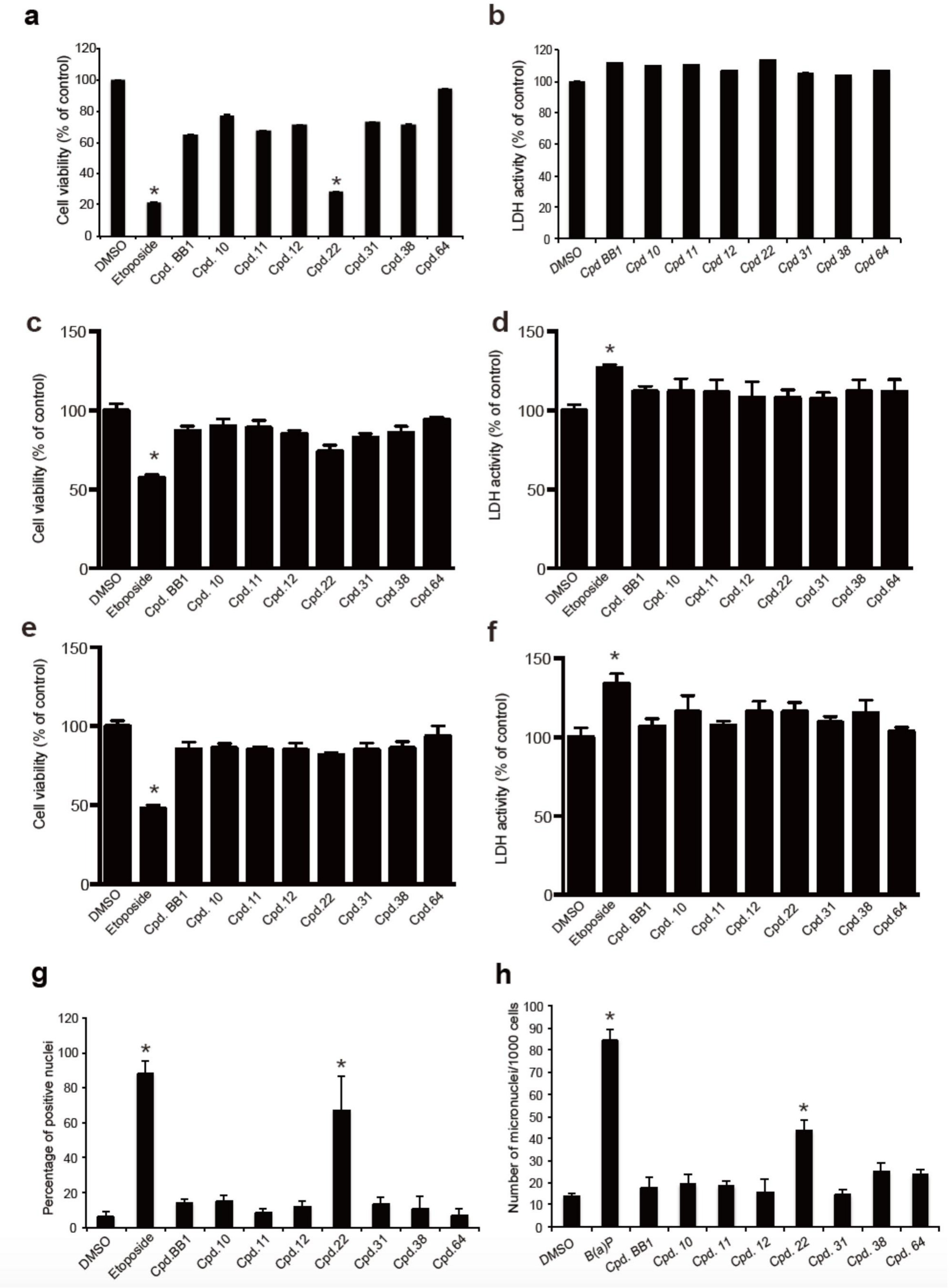

Supplementary Figure 3. Cytotoxicity and genotoxicity of the compounds.

(**a**) MTT assay showing the cytotoxicity of the compounds in HepG2 cells. Etoposide was used as a positive control.

(**b**) LDH assay showing the toxic effect of the compounds in primary neurons.

(**c**) MTT assay showing the cytotoxicity of the compounds in HEK293 cells

(**d**) LDH assay showing the toxic effect of the compounds in HEK293 cells.

(**e**) MTT assay showing the cytotoxicity of the compounds in Pala cells

(**f**) LDH assay showing the toxic effect of the compounds in Pala cells.

(**g**) COMET assay results. Etoposide was used as a positive control.

(**h**) Micronucleus assay results. Benzo(α)pyrene (B(a)p) was used as a positive control. All data represent mean ± s.d. of three independent experiments.

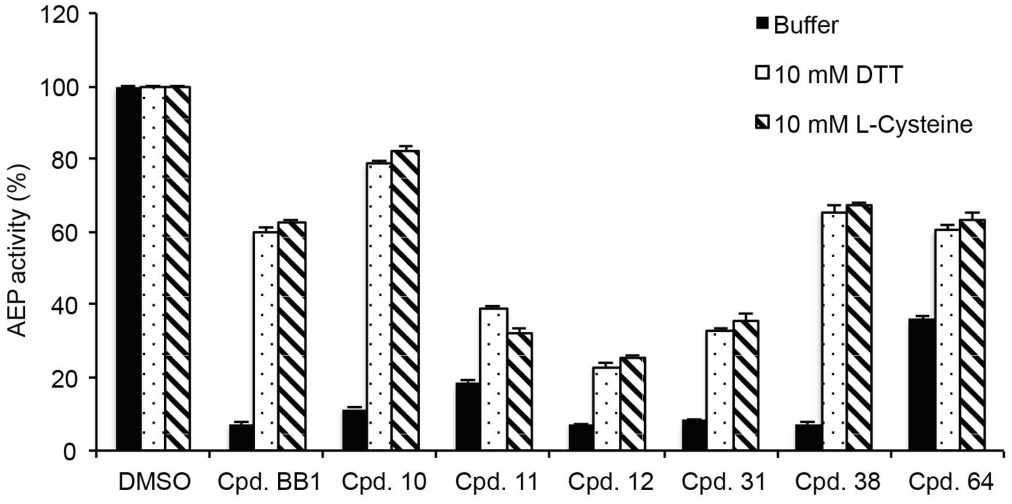

Supplementary Figure 4. DTT and L-Cysteine Reversibility of the inhibitors towards δ-secretase.

δ-secretase was reacted with the specified inhibitor, after 15 min 10 mM DTT or L-cysteine was added to the reaction and the fluorescence signal was read for an additional 15 min. At the end of the second 15 min incubation, the percentage of product formed in the presence of each compound was determined in comparison to the DMSO control reaction. Data represent mean ± s.d. of three independent experiments.

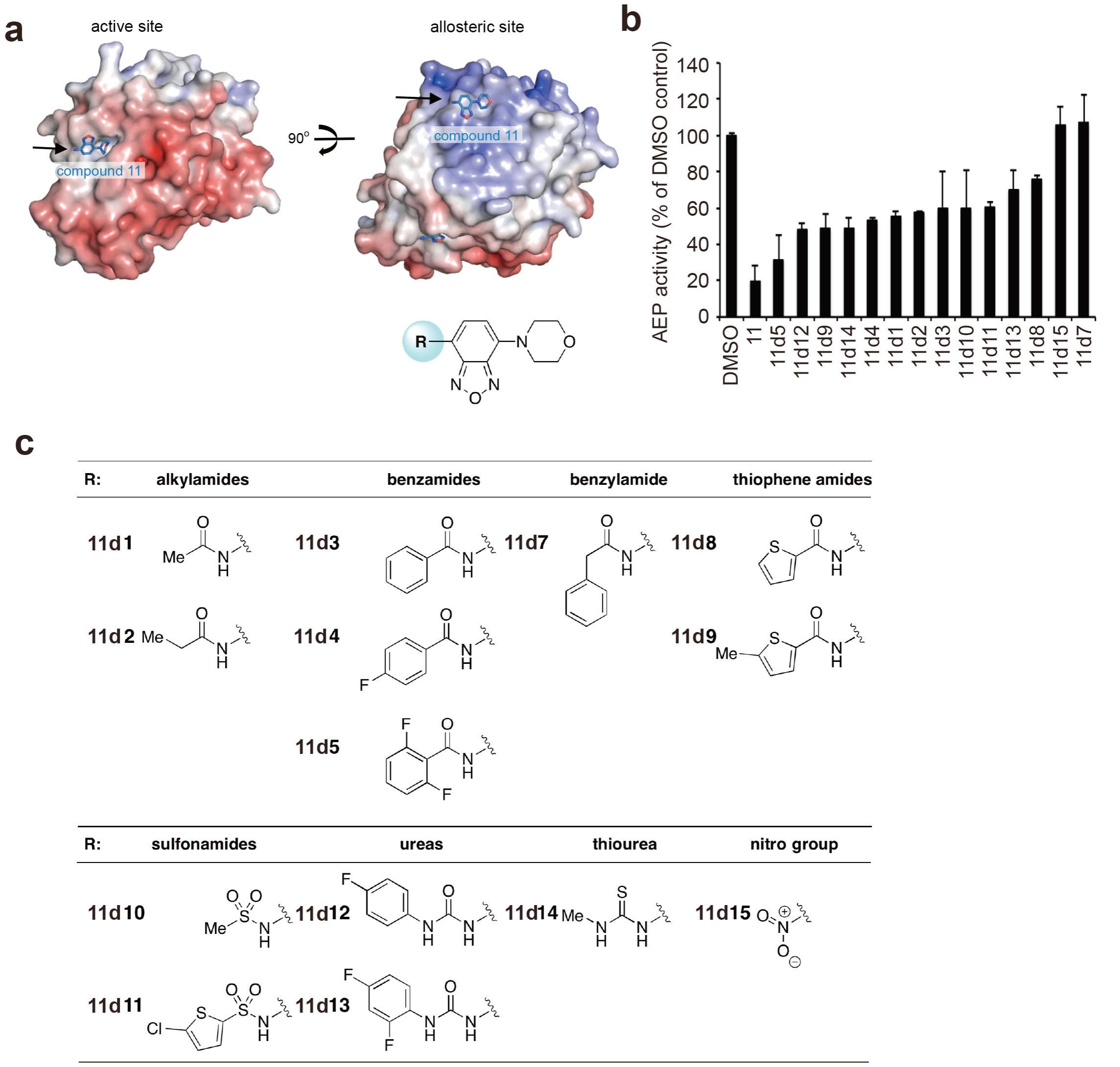

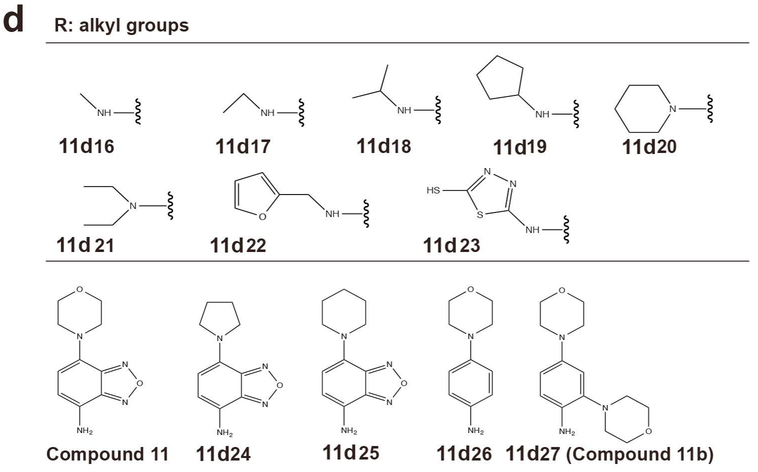

e

Compound 11 synthetic derivatives	IC50 (μM)
11d16	0.124
11d17	0.135
11d18	0.155
11d19	0.114
11d20	0.645
11d21	0.135
11d22	0.237
11d23	0.017
11d24	1.640
11d25	5.410
11d26	20.530
11d27	0.110

Supplementary Figure 5. Structure-Activity Relationship (SAR) Assay of compound 11.

(**a**) Color-coded electrostatic surface potential of δ-secretase (red: negative charge, blue: positive charge). Compound 11 is shown in in blue sticks.

(**b-e**) SAR study of compound 11. In panel e, the IC50s indicate the IC50 against purified AEP as in Figure 1.

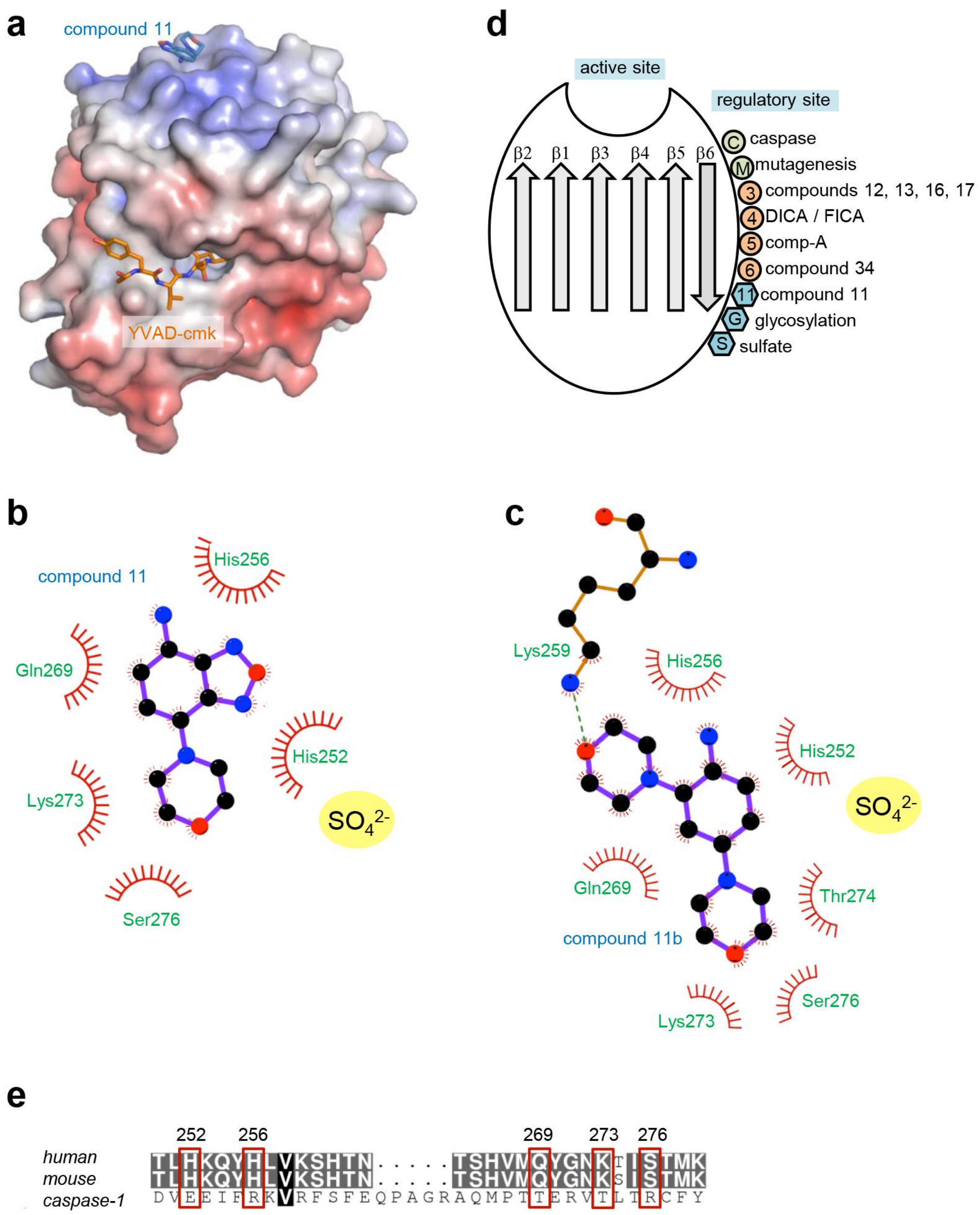

Supplementary Figure 6. The binding site of compound 11 on δ-secretase is close to allosteric regulatory sites in caspases.

(**a**) Color-coded electrostatic surface potential of δ-secretase (red: negative charge, blue: positive charge) calculated at pH 7.0. The covalent YVAD-cmk inhibitor labeling the active site is shown in orange sticks, compound 11 in blue sticks.

(**b-c**) 2d interaction diagrams of compound 11 (b) and compound 11 b (c) with δ-secretase. The diagrams were prepared using the LigPlot$^+$ software. Residues on δ-secretase interacting with compound 11(b) are labelled in green; a sulfate ion is indicated as yellow sphere.

(**d**) (Small molecule) binding sites on caspases and legumain cluster around the β6 strand. The

β6 strand serves as the dimerization interface in caspases (c). Consistently, selected mutagenesis in the dimer interface leads to allosteric inhibition of caspases (M). Several activity modulating molecules have been identified which bind close to β6 (3 – 6). Likewise, we find compound 11 binding close to the β6 strand on legumain (11). Additionally, a sulfate ion (S) and two glycosylation sites (Asn167 and Asn272; g) are located near β6 in legumain.

(**e**) Sequence alignment showing the conservation of the allosteric compound 11(b) binding site in human (Q99538) and mouse (NP_776526.1) δ-secretase. Interacting residues are labelled. The sequence numbering is based on human legumain. Interacting residues are not conserved in human caspase-1 (P29466). The alignment was created with ClustalW and modified with Aline.

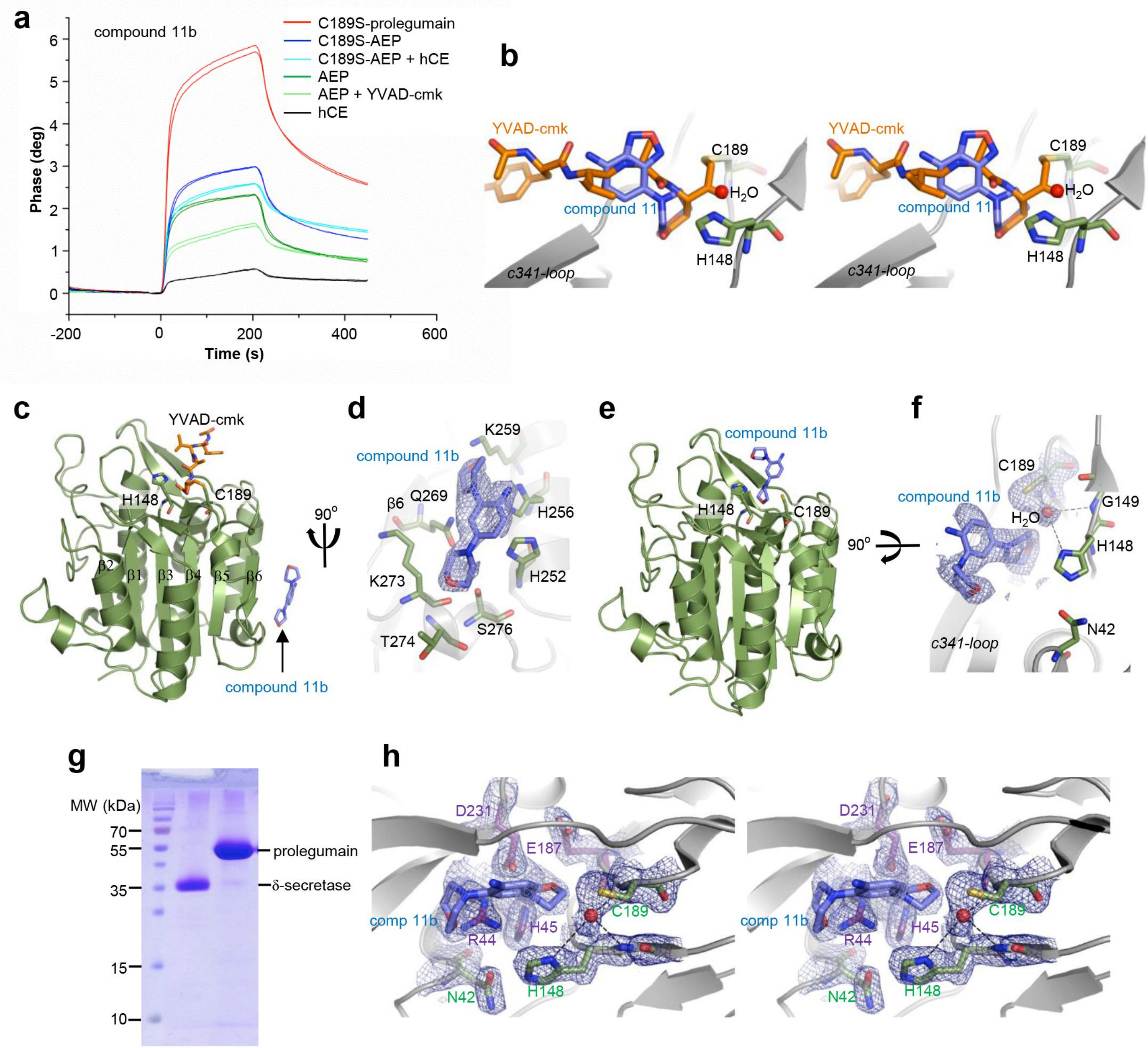

Supplementary Figure 7. Binding of compound 11b to δ-secretase.

(**a**) Similar to compound 11, compound 11b is binding to active site liganded and free δ-secretase. Compound 11b was immobilized on a sam®5BLUE biosensor chip and binding of C189S-prolegumain (red curves), in trans activated C189S-legumain only (dark blue curves) and complexed to cystatin E (light blue curves), δ-secretase (dark green curves) and δ-secretase covalently inhibited with YVAD-chloromethyleketone (δ-secretase + YVAD-cmk, light green curves), was tested at pH 5.0. Cystatin E (black curves) served as a control to test for unspecific binding.

(**b**) Binding of compound 11 to the δ-secretase active site is substrate-like. Stereo-view on the

active site of YVAD-cmk and compound 11 bound δ-secretase. Compound 11 is mimicking a substrate in P1 and P2 position. Furthermore, a water molecule is occupying the oxyanion pocket. Thereby, compound 11 together with the oxyanion generates a transition state analog.

(**c**) Compound 11b binds to the δ-secretase allosteric site of YVAD-cmk inhibited δ-secretase. The active site is labelled with the covalent YVAD-cmk inhibitor and shown in orange sticks, the catalytic Cys189 and His148 in green sticks and compound 11b in blue sticks.

(**d**) Zoom-in view on the compound 11b binding site.

(**e**) Compound 11b binds to the δ-secretase active site, similar to compound 11.

(**f**) Zoom-in view on the active site. The morpholino group binds into δ-secretase's S1-pocket. Furthermore, the oxyanion-pocket, formed by Cys189, Gly149 and His148, is also occupied in the structure. The electron density ($2F_{obs} - F_{calc}$) defining compound 11b is contoured at 1σ over the mean. Interacting residues on δ-secretase are shown as green sticks.

(**g**) SDS-PAGE gel of human pro-δ-secretase and pH-activated δ-secretase used for crystallization experiments confirming its purity of > 95%.

(**h**) Stereo-view on the active site of δ-secretase in complex with compound 11b. The electron density ($2F_{obs} - F_{calc}$) defining S1-specificity residues (purple sticks), catalytic residues (green sticks) and compound 11b (blue sticks) is contoured at 1σ over the mean.

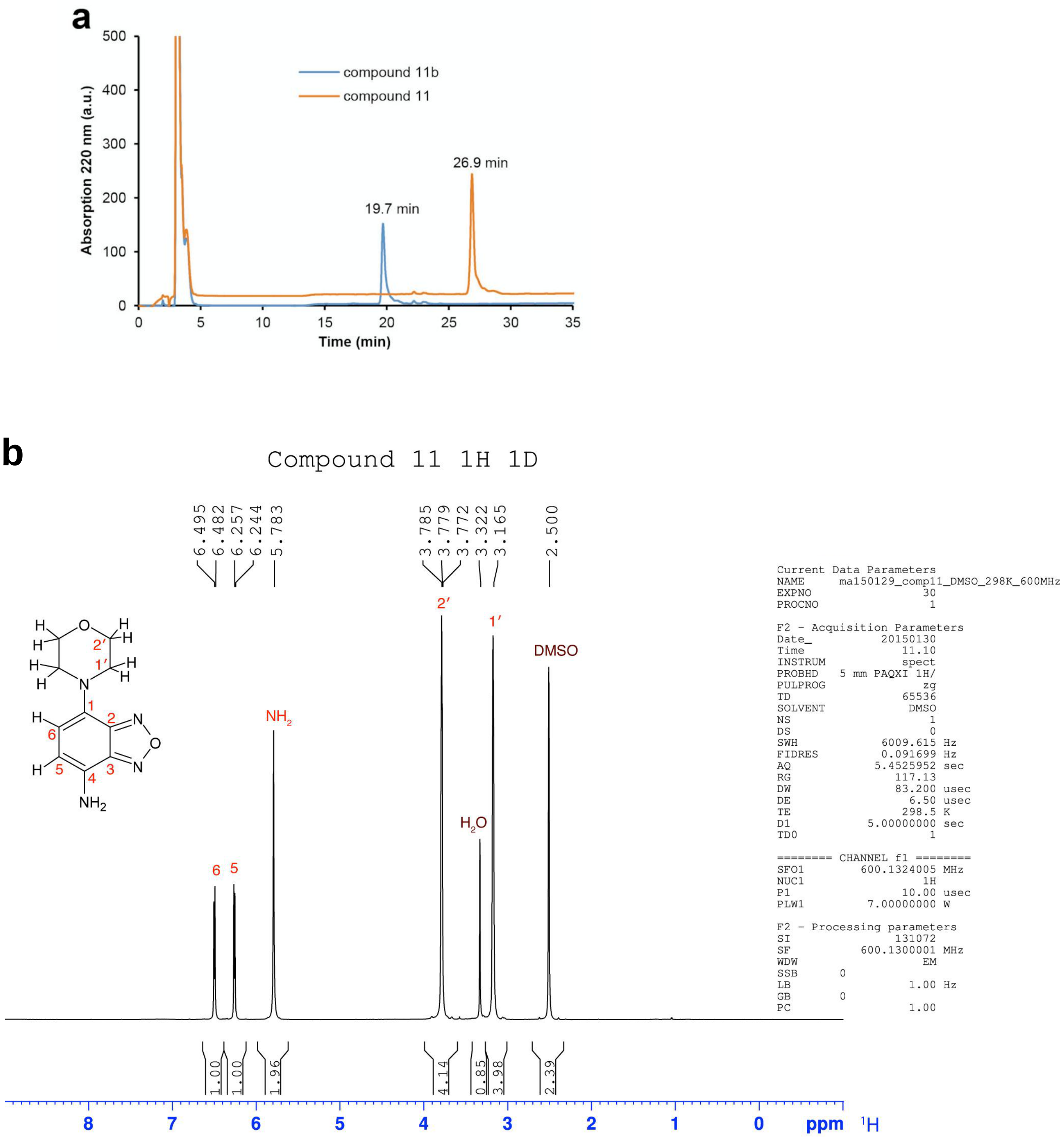

a
Absorption 220 nm (a.u.)
500
400
300
200
100
0
0
5
10
15
20
25
30
35
Time (min)
compound 11b
compound 11
26.9 min
19.7 min
b
Compound 11 1H 1D
6.495
6.482
6.257
6.244
5.783
3.785
3.779
3.772
3.322
3.165
2.500
2′
1′
DMSO
NH2
H2O
6
5
1.00
1.00
1.96
4.14
0.85
3.98
2.39
8
7
6
5
4
3
2
1
0
ppm
1H
Current Data Parameters
NAME ma150129_comp11_DMSO_298K_600MHz
EXPNO 30
PROCNO 1
F2 - Acquisition Parameters
Date_ 20150130
Time 11.10
INSTRUM spect
PROBHD 5 mm PAQXI 1H/
PULPROG zg
TD 65536
SOLVENT DMSO
NS 1
DS 0
SWH 6009.615 Hz
FIDRES 0.091699 Hz
AQ 5.4525952 sec
RG 117.13
DW 83.200 usec
DE 6.50 usec
TE 298.5 K
D1 5.00000000 sec
TD0 1
======== CHANNEL f1 ========
SFO1 600.1324005 MHz
NUC1 1H
P1 10.00 usec
PLW1 7.00000000 W
F2 - Processing parameters
SI 131072
SF 600.1300001 MHz
WDW EM
SSB 0
LB 1.00 Hz
GB 0
PC 1.00

c

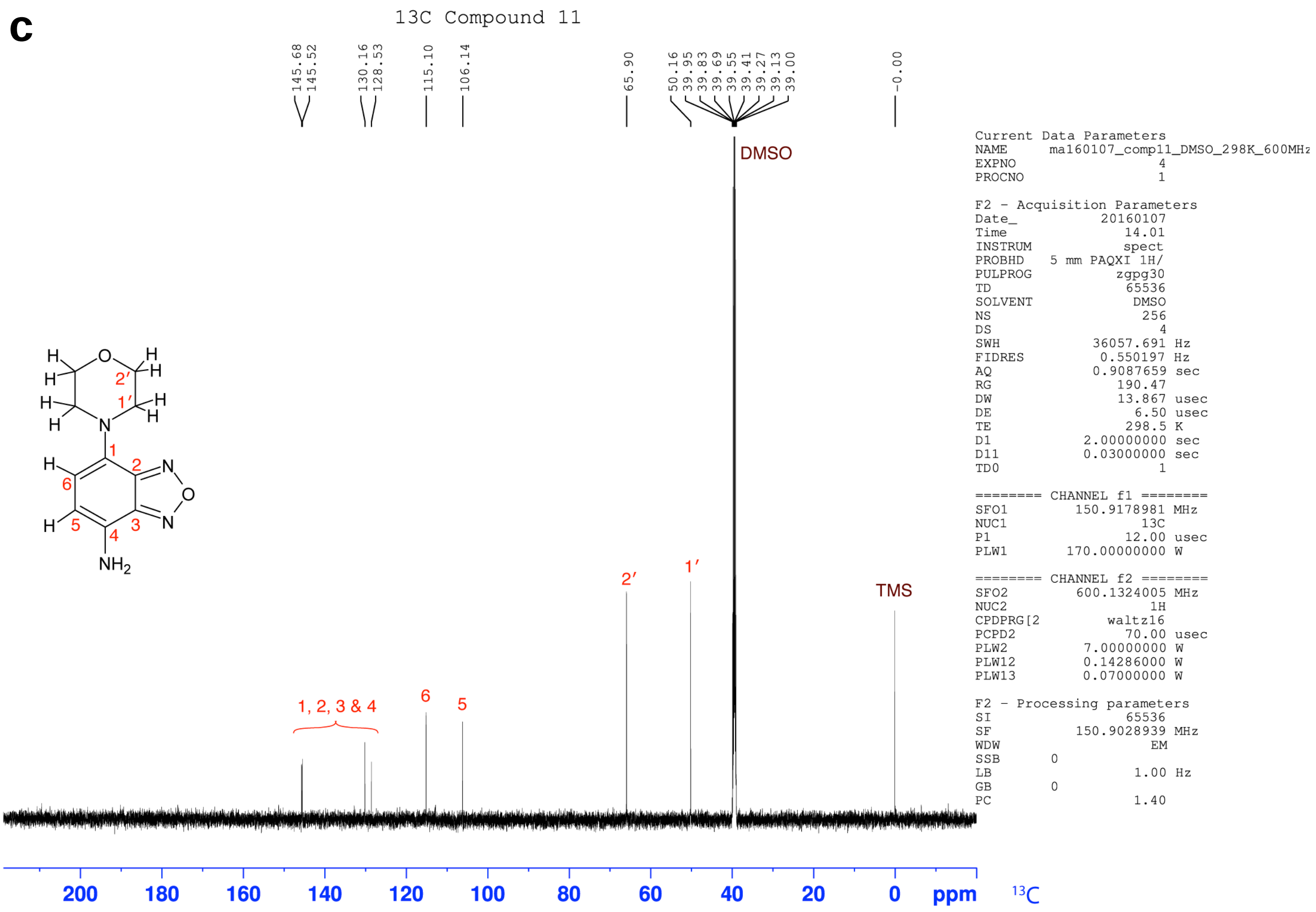
13C Compound 11
145.68
145.52
130.16
128.53
115.10
106.14
65.90
50.16
39.95
39.83
39.69
39.55
39.41
39.27
39.13
39.00
-0.00
DMSO
2′
1′
TMS
1, 2, 3 & 4
6
5
NH2
200
180
160
140
120
100
80
60
40
20
0
ppm
13C
Current Data Parameters
NAME ma160107_comp11_DMSO_298K_600MHz
EXPNO 4
PROCNO 1
F2 - Acquisition Parameters
Date_ 20160107
Time 14.01
INSTRUM spect
PROBHD 5 mm PAQXI 1H/
PULPROG zgpg30
TD 65536
SOLVENT DMSO
NS 256
DS 4
SWH 36057.691 Hz
FIDRES 0.550197 Hz
AQ 0.9087659 sec
RG 190.47
DW 13.867 usec
DE 6.50 usec
TE 298.5 K
D1 2.00000000 sec
D11 0.03000000 sec
TD0 1
======== CHANNEL f1 ========
SFO1 150.9178981 MHz
NUC1 13C
P1 12.00 usec
PLW1 170.00000000 W
======== CHANNEL f2 ========
SFO2 600.1324005 MHz
NUC2 1H
CPDPRG[2 waltz16
PCPD2 70.00 usec
PLW2 7.00000000 W
PLW12 0.14286000 W
PLW13 0.07000000 W
F2 - Processing parameters
SI 65536
SF 150.9028939 MHz
WDW EM
SSB 0
LB 1.00 Hz
GB 0
PC 1.40

d

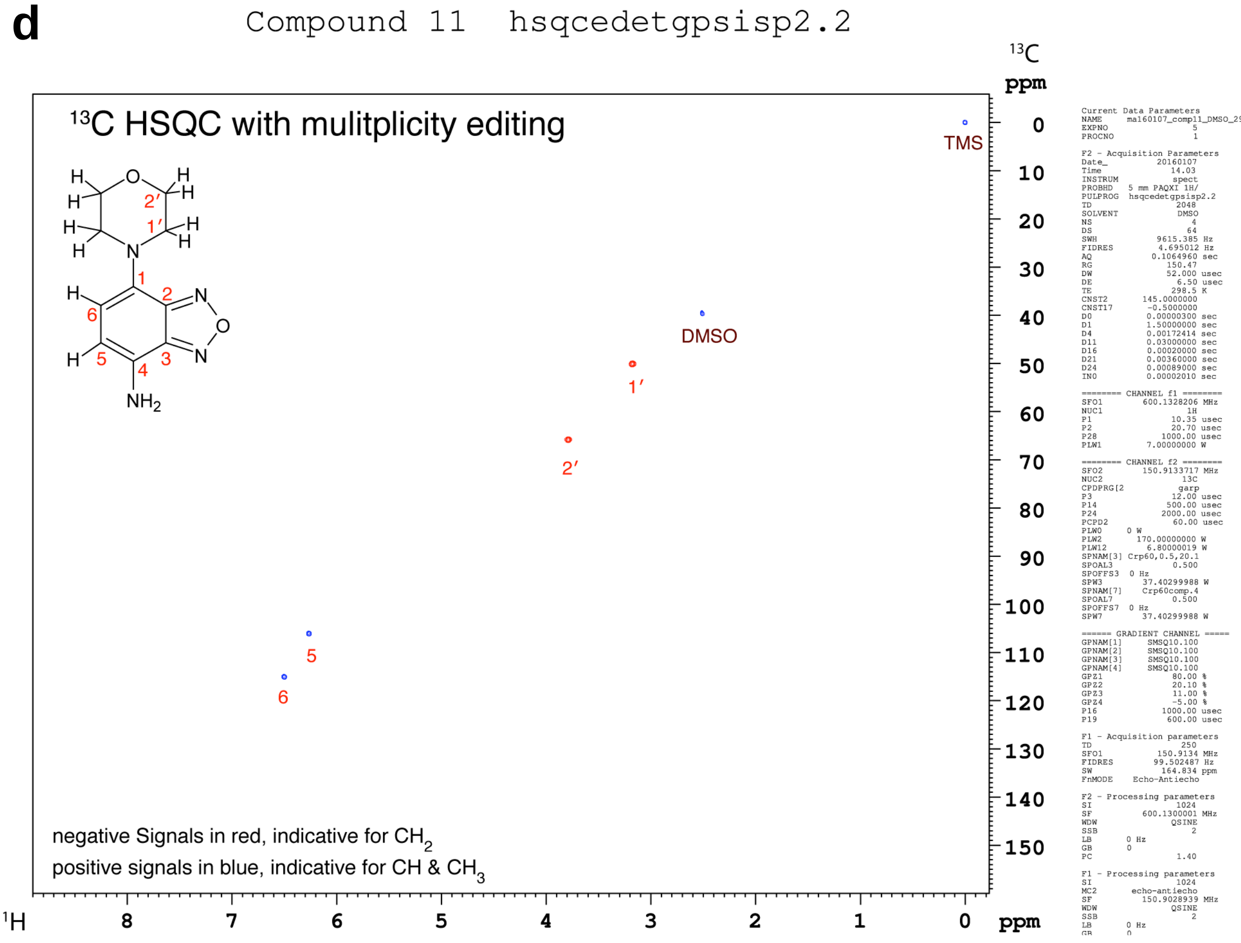
Compound 11 hsqcedetgpsisp2.2
13C HSQC with mulitplicity editing
TMS
DMSO
1′
2′
5
6
NH2
negative Signals in red, indicative for CH2
positive signals in blue, indicative for CH & CH3
13C
ppm
0
10
20
30
40
50
60
70
80
90
100
110
120
130
140
150
1H
8
7
6
5
4
3
2
1
0
ppm
Current Data Parameters
NAME ma160107_comp11_DMSO_298K_600MHz
EXPNO 5
PROCNO 1

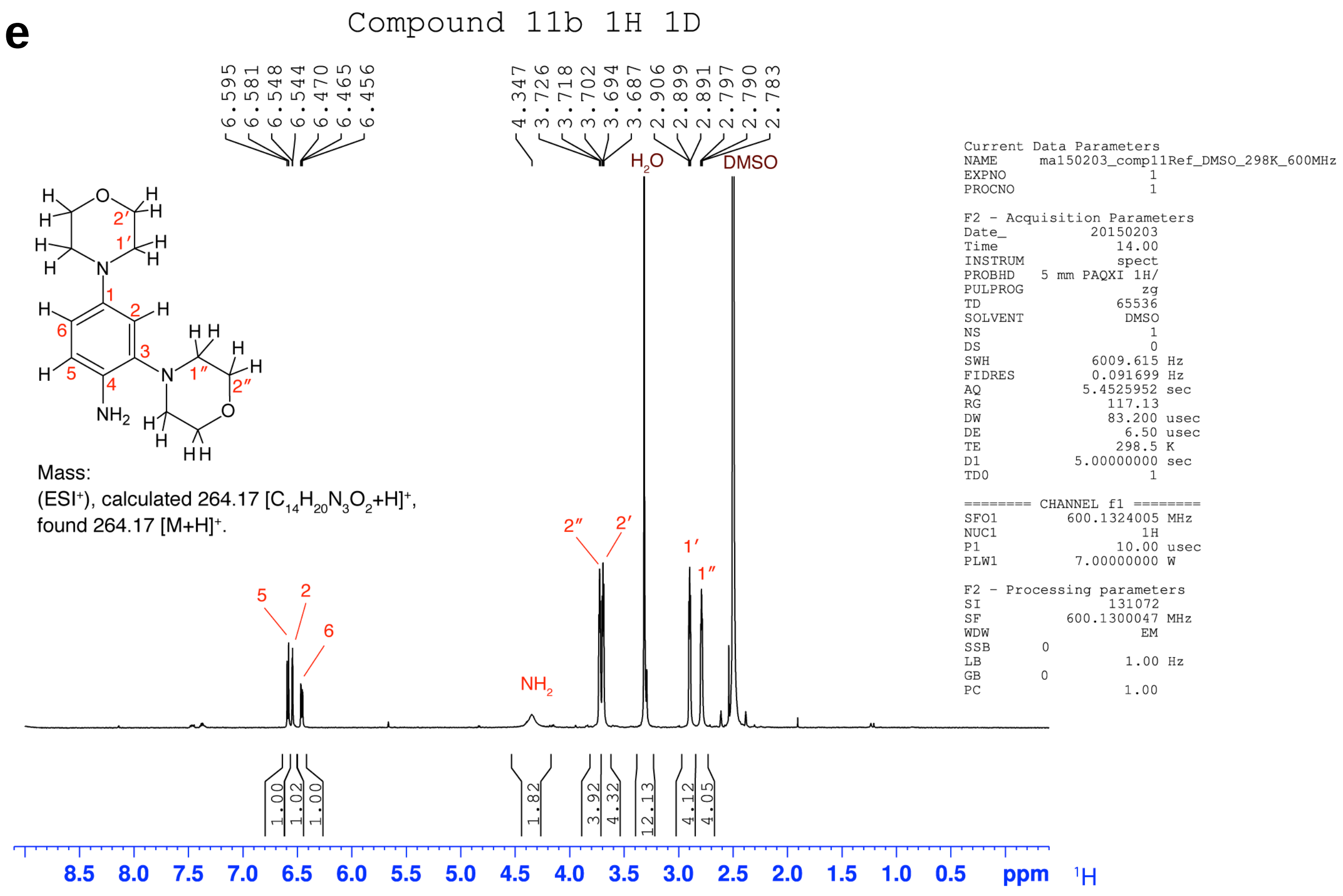
e
Compound 11b 1H 1D
6.595 6.581 6.548 6.544 6.470 6.465 6.456
4.347 3.726 3.718 3.702 3.694 3.687 2.906 2.899 2.891 2.797 2.790 2.783
H2O
DMSO
Mass:
(ESI+), calculated 264.17 [C14H20N3O2+H]+,
found 264.17 [M+H]+.
5 2 6
NH2
2″ 2′
1′ 1″
1.00 1.02 1.00 1.82 3.92 4.32 12.13 4.12 4.05
8.5 8.0 7.5 7.0 6.5 6.0 5.5 5.0 4.5 4.0 3.5 3.0 2.5 2.0 1.5 1.0 0.5 ppm
1H
Current Data Parameters
NAME ma150203_comp11Ref_DMSO_298K_600MHz
EXPNO 1
PROCNO 1
F2 - Acquisition Parameters
Date_ 20150203
Time 14.00
INSTRUM spect
PROBHD 5 mm PAQXI 1H/
PULPROG zg
TD 65536
SOLVENT DMSO
NS 1
DS 0
SWH 6009.615 Hz
FIDRES 0.091699 Hz
AQ 5.4525952 sec
RG 117.13
DW 83.200 usec
DE 6.50 usec
TE 298.5 K
D1 5.00000000 sec
TD0 1
======== CHANNEL f1 ========
SFO1 600.1324005 MHz
NUC1 1H
P1 10.00 usec
PLW1 7.00000000 W
F2 - Processing parameters
SI 131072
SF 600.1300047 MHz
WDW EM
SSB 0
LB 1.00 Hz
GB 0
PC 1.00

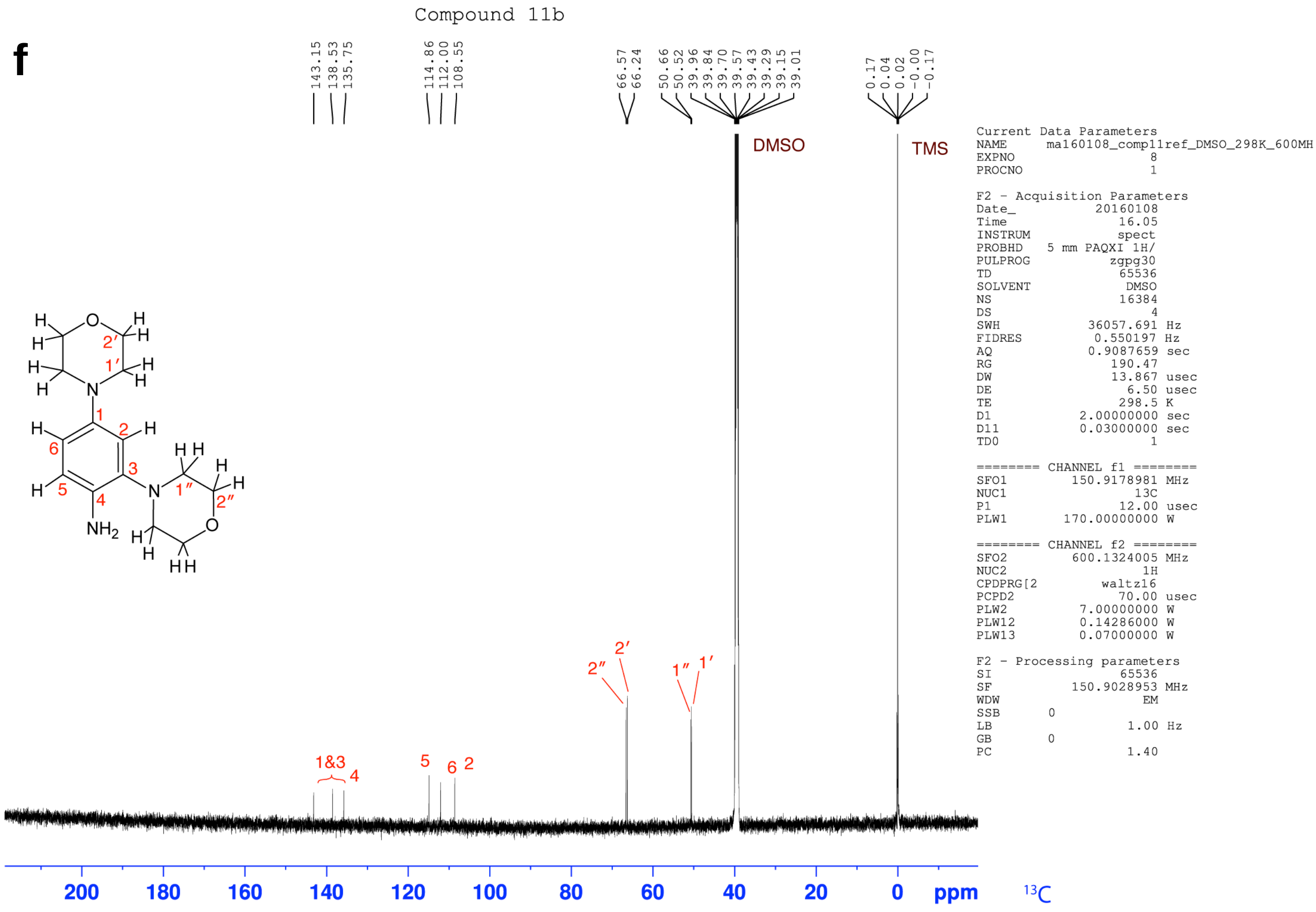
f
Compound 11b
143.15 138.53 135.75
114.86 112.00 108.55
66.57 66.24
50.66 50.52 39.96 39.84 39.70 39.57 39.43 39.29 39.15 39.01
0.17 0.04 0.02 -0.00 -0.17
DMSO
TMS
1&3 4
5 6 2
2″ 2′
1″ 1′
200 180 160 140 120 100 80 60 40 20 0 ppm
13C
Current Data Parameters
NAME ma160108_comp11ref_DMSO_298K_600MH
EXPNO 8
PROCNO 1
F2 - Acquisition Parameters
Date_ 20160108
Time 16.05
INSTRUM spect
PROBHD 5 mm PAQXI 1H/
PULPROG zgpg30
TD 65536
SOLVENT DMSO
NS 16384
DS 4
SWH 36057.691 Hz
FIDRES 0.550197 Hz
AQ 0.9087659 sec
RG 190.47
DW 13.867 usec
DE 6.50 usec
TE 298.5 K
D1 2.00000000 sec
D11 0.03000000 sec
TD0 1
======== CHANNEL f1 ========
SFO1 150.9178981 MHz
NUC1 13C
P1 12.00 usec
PLW1 170.00000000 W
======== CHANNEL f2 ========
SFO2 600.1324005 MHz
NUC2 1H
CPDPRG[2 waltz16
PCPD2 70.00 usec
PLW2 7.00000000 W
PLW12 0.14286000 W
PLW13 0.07000000 W
F2 - Processing parameters
SI 65536
SF 150.9028953 MHz
WDW EM
SSB 0
LB 1.00 Hz
GB 0
PC 1.40

g

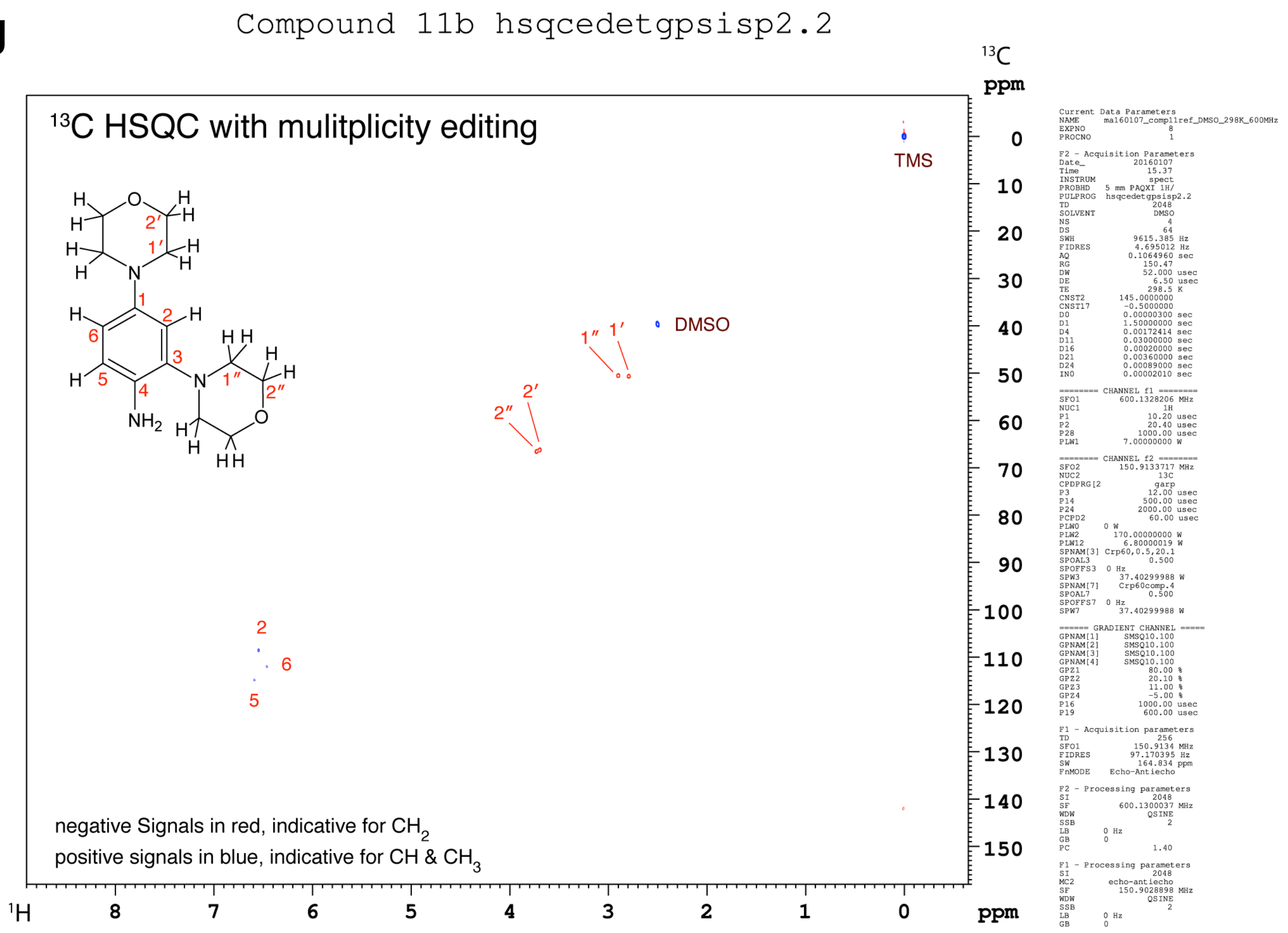
Compound 11b hsqcedetgpsisp2.2
13C HSQC with mulitplicity editing
TMS
DMSO
negative Signals in red, indicative for CH2
positive signals in blue, indicative for CH & CH3
13C ppm
1H
ppm
Current Data Parameters

h

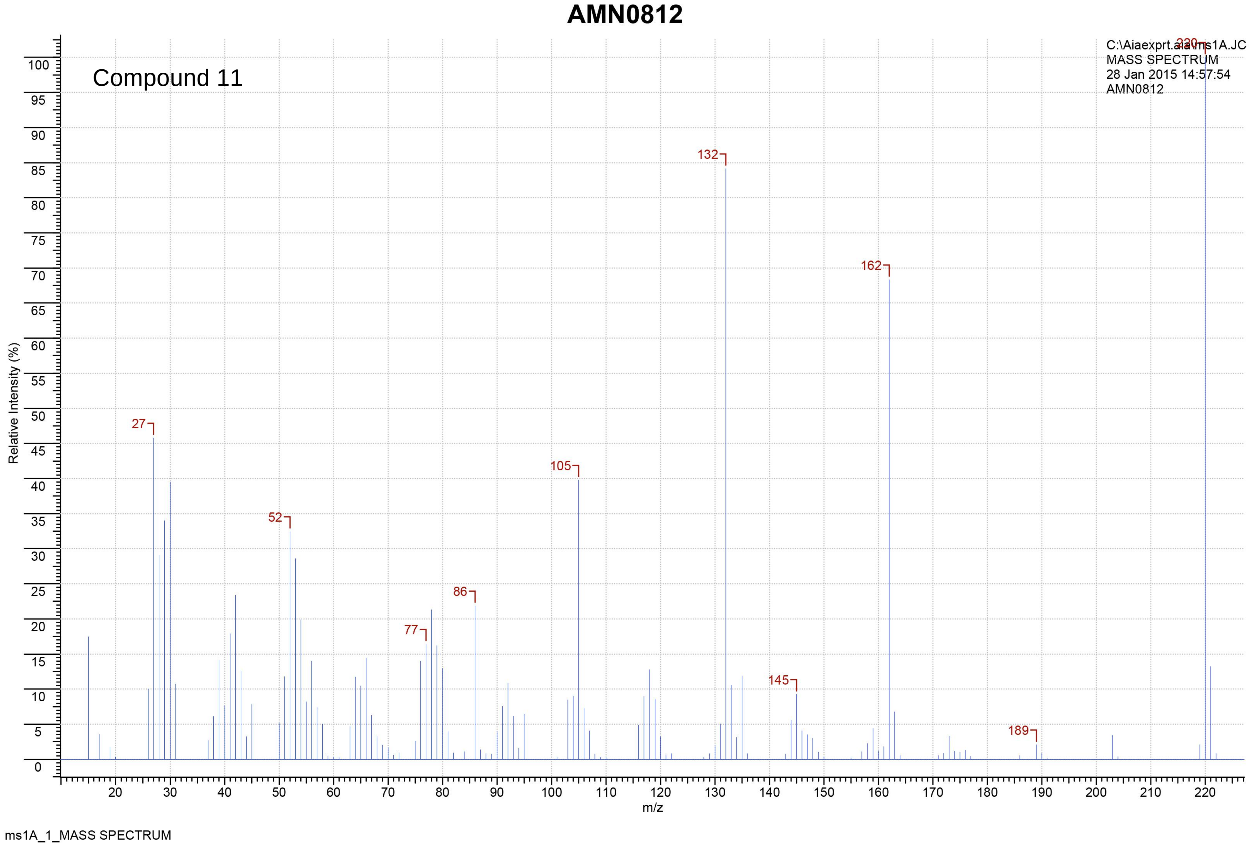
AMN0812
Compound 11
C:\Aiaexprt.aia\ms1A.JC
MASS SPECTRUM
28 Jan 2015 14:57:54
AMN0812
Relative Intensity (%)
m/z
ms1A_1_MASS SPECTRUM

i

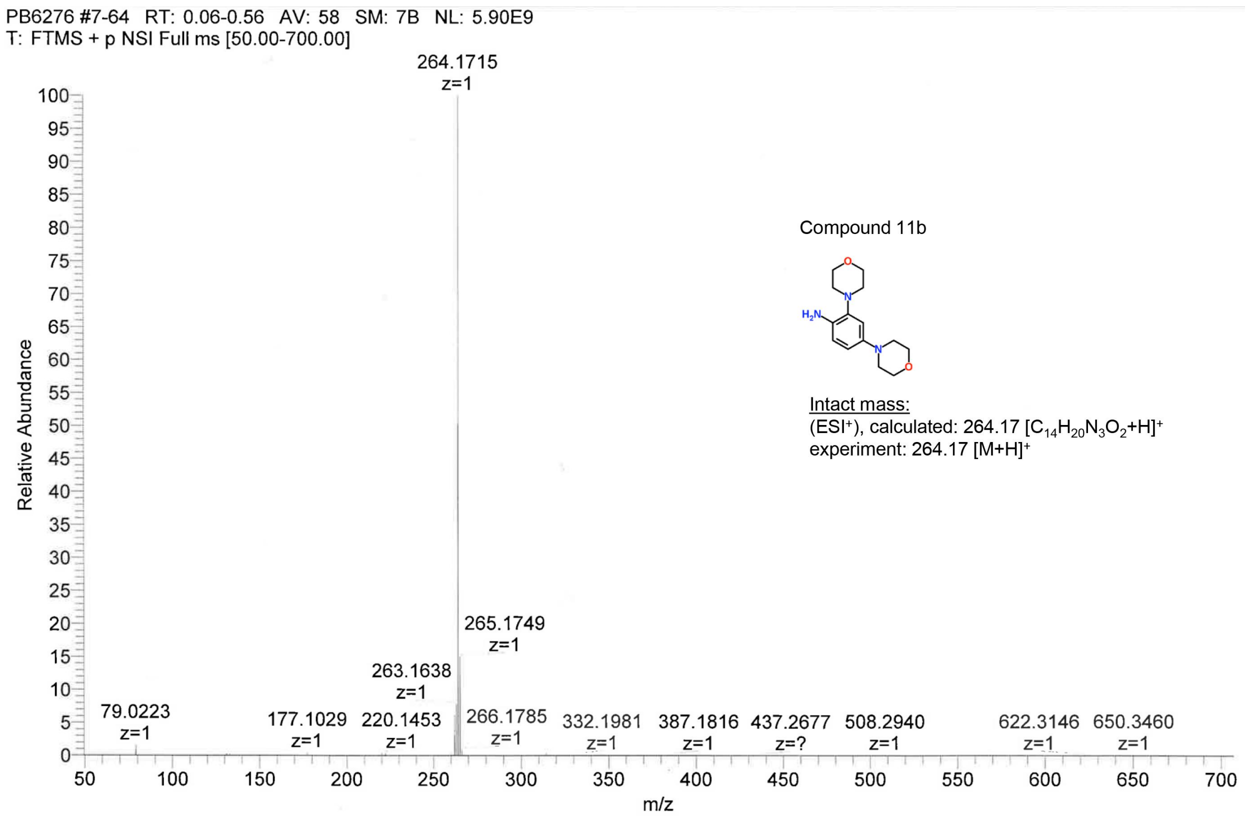

Supplementary Figure 8. Identity and homogeneity of compounds 11 & 11b.

(**a**) Reversed phase HPLC chromatograms (C18 column) of compound 11b and 11 were detected at 220 nm absorption, resulting in unimodal elution peaks at 19.7 and 26.9 min, respectively.

(**b-d**) The ^{1}H, ^{13}C and two-Dimensional NMR of compound 11.

(**e-g**) The ^{1}H, ^{13}C and two-Dimensional NMR of compound 11b.

(**h-i**) MS spectra of compound 11 and 11b.

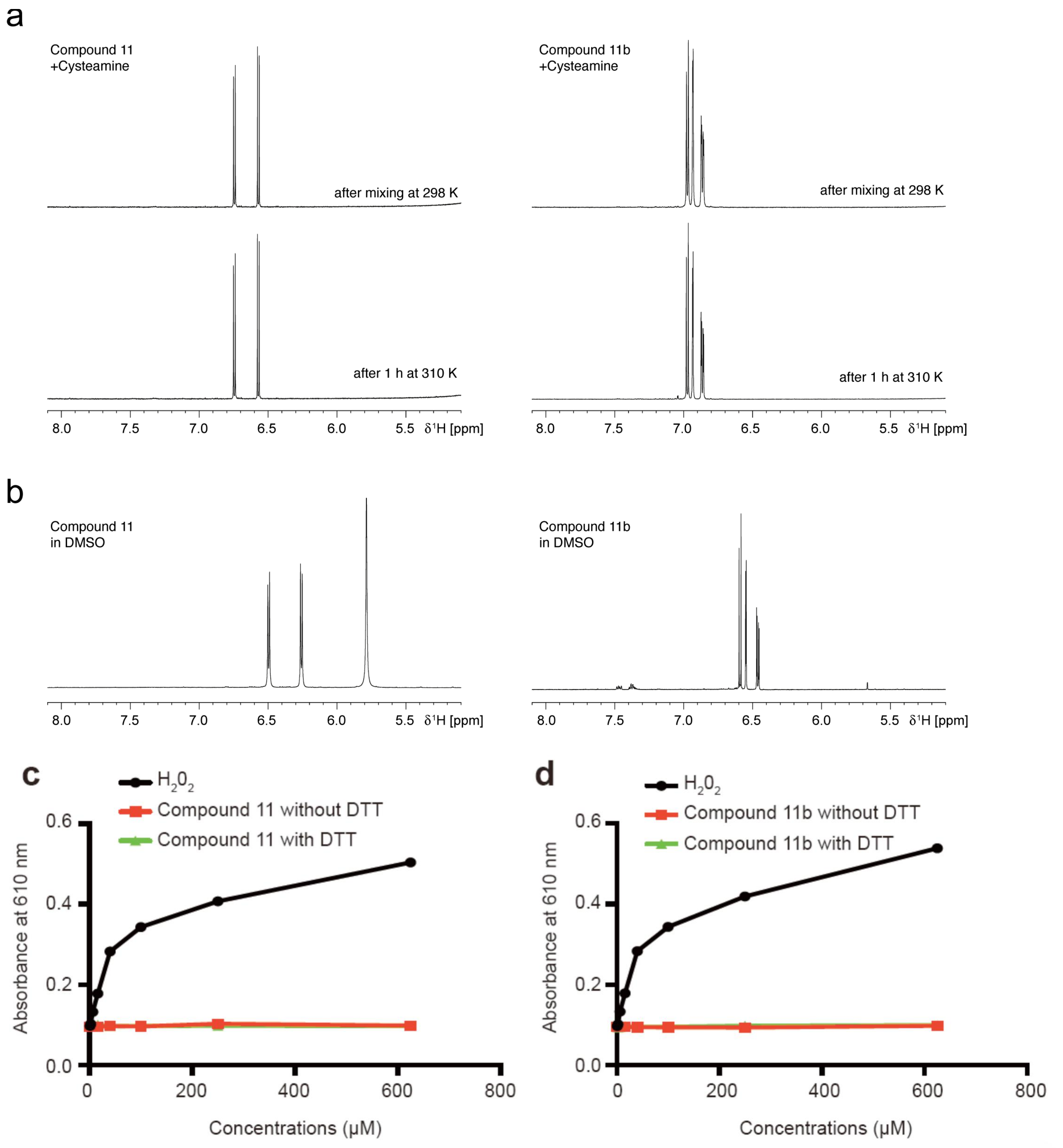

Supplementary Figure 9: Stability and redox potential of compounds 11 and 11b.

(**a-b**) NMR spectroscopy showing that both compounds 11 and 11b are stable in the presence and absence of cysteamine.

(**c-d**) The redox potential of compounds 11 and 11b determined with horseradish peroxidase-phenol red assay.

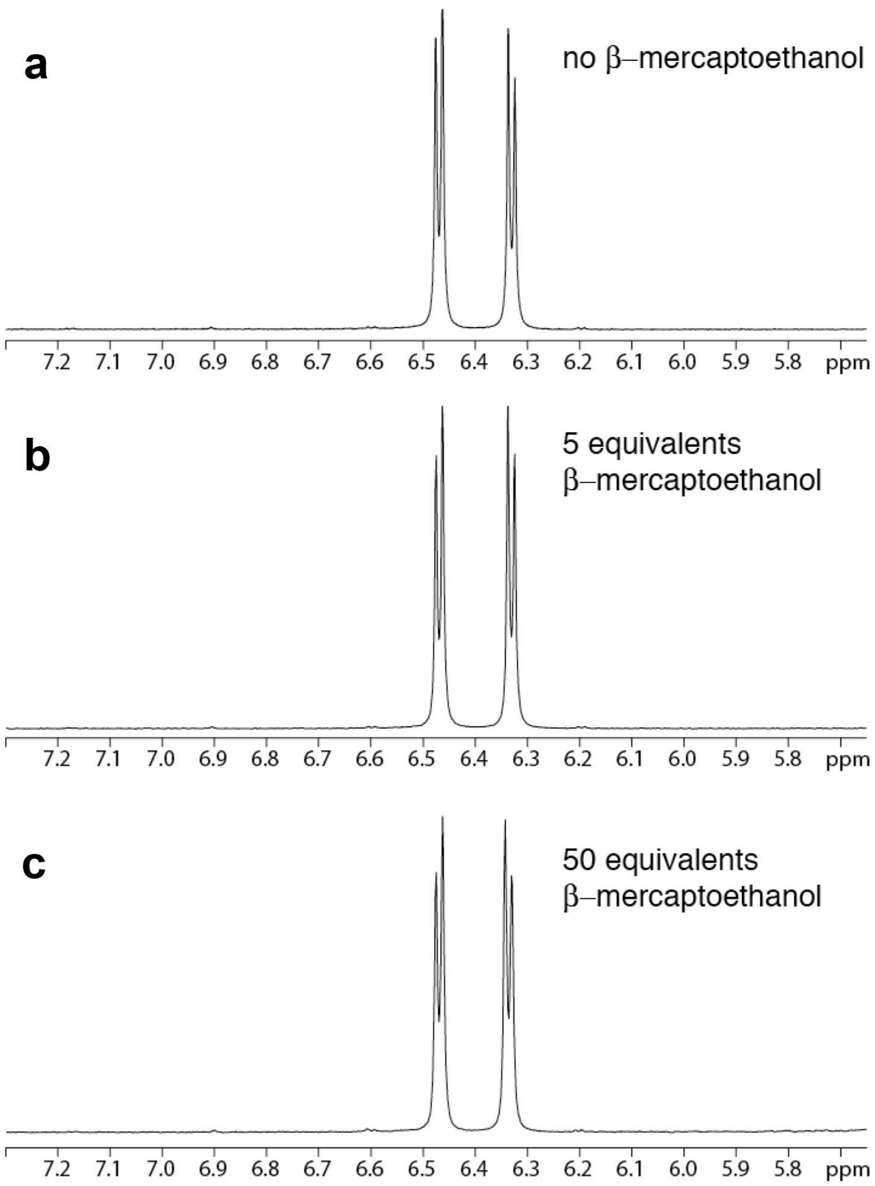

Supplementary Figure 10: Influence of β-mercaptoethanol on the NMR spectrum of compound 11 (25 mM), measured in methanol d4 and illustrated by the aromatic signals. 1D ^{1}H spectrum in absence (**a**), 5 equivalents (**b**) and 50 equivalents of β-mercaptoethanol (**c**).

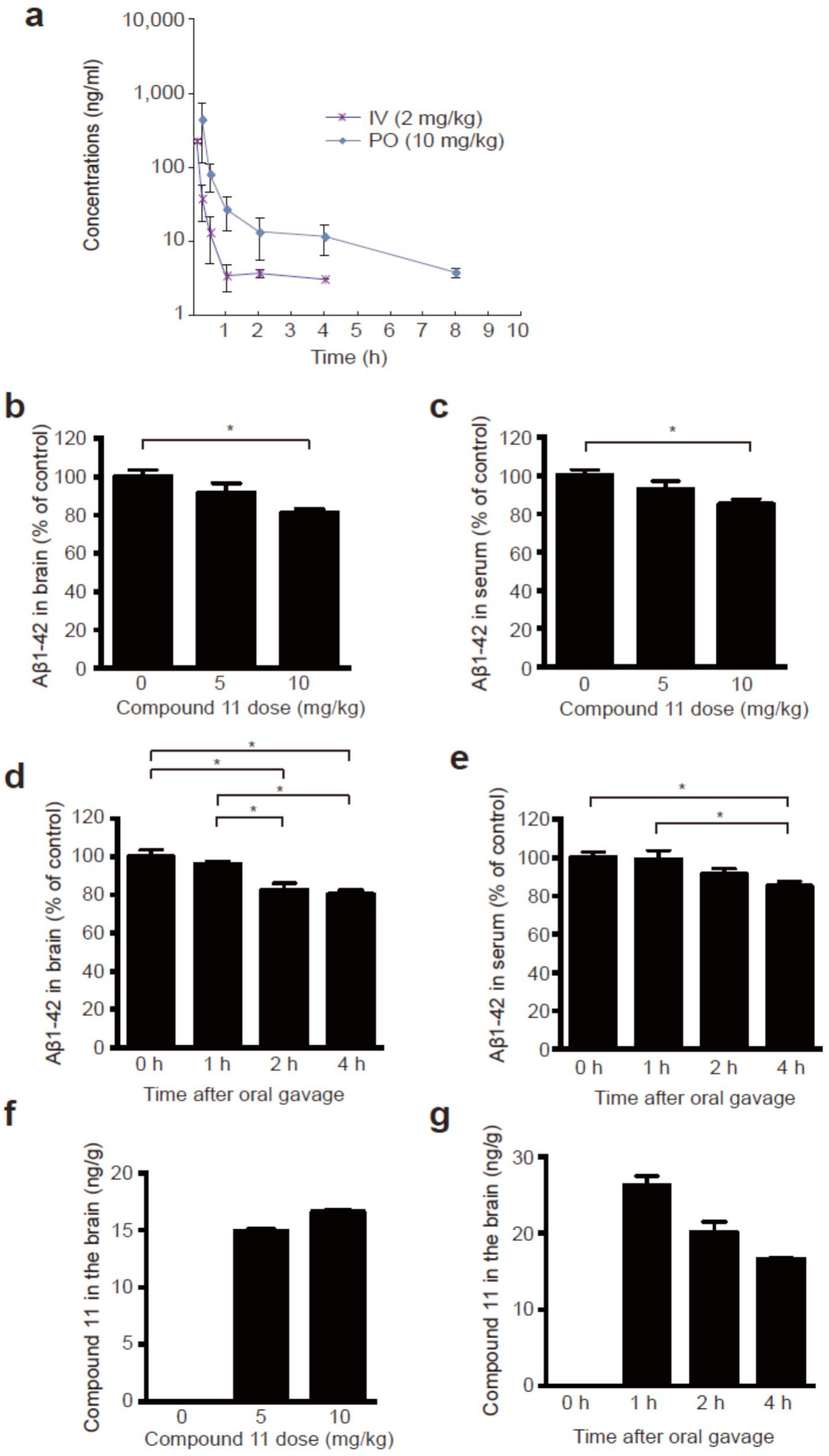
a
Concentrations (ng/ml)
10,000
1,000
100
10
1
IV (2 mg/kg)
PO (10 mg/kg)
1 2 3 4 5 6 7 8 9 10
Time (h)
b
Aβ1-42 in brain (% of control)
0 5 10
Compound 11 dose (mg/kg)
c
Aβ1-42 in serum (% of control)
0 5 10
Compound 11 dose (mg/kg)
d
Aβ1-42 in brain (% of control)
0 h 1 h 2 h 4 h
Time after oral gavage
e
Aβ1-42 in serum (% of control)
0 h 1 h 2 h 4 h
Time after oral gavage
f
Compound 11 in the brain (ng/g)
0 5 10
Compound 11 dose (mg/kg)
g
Compound 11 in the brain (ng/g)
0 h 1 h 2 h 4 h
Time after oral gavage

Supplementary Figure 11. In vivo pharmacokinetic and pharmacodynamic study of compound 11 in mice.

(**a**) Pharmacokinetic study of compound 11. Two months old CD-1 male mice were orally administrated 10 mg kg^{-1} of compound 11 or i.v. injected 2 mg kg^{-1} of compound 11. The serum was collected at indicated time points with each group of 3 mice (mean ± s.d.). The lowest limit of quantification of compound 11 in plasma was 2 ng ml^{-1}.

(**b-c**) Compound 11 time-dependently decreases Aβ concentration in brain and serum. 5XFAD mice were orally administrated with 10 mg kg^{-1} compound 11. 1 h, 2 h, and 4 h after administration, the concentrations of Aβ in the brain and serum were determined using ELISA.

(**d-e**) Compound 11 dose-dependently decreases Aβ concentration in brain and serum. 5XFAD mice were were orally administrated vehicle, 5 mg kg^{-1}, or 10 mg kg^{-1} compound 11. 4 h later, the concentrations of Aβ in the brain and serum were determined using ELISA (mean ± s.e.m.; n = 10 mice per group).

(**f**) Pharmacokinetics of compound 11 in 5XFAD mice. 5XFAD mice were orally administrated with vehicle, 5 mg kg^{-1}, or 10 mg kg^{-1} compound 11. 4 h after administration, the concentrations of compound 11 in the brain were determined.

(**g**) Pharmacokinetics of compound 11 in 5XFAD mice. 5XFAD mice were orally administrated with 10 mg kg^{-1} compound 11. 1 h, 2 h, and 4 h after administration, the concentrations of compound 11 in the brain were determined.

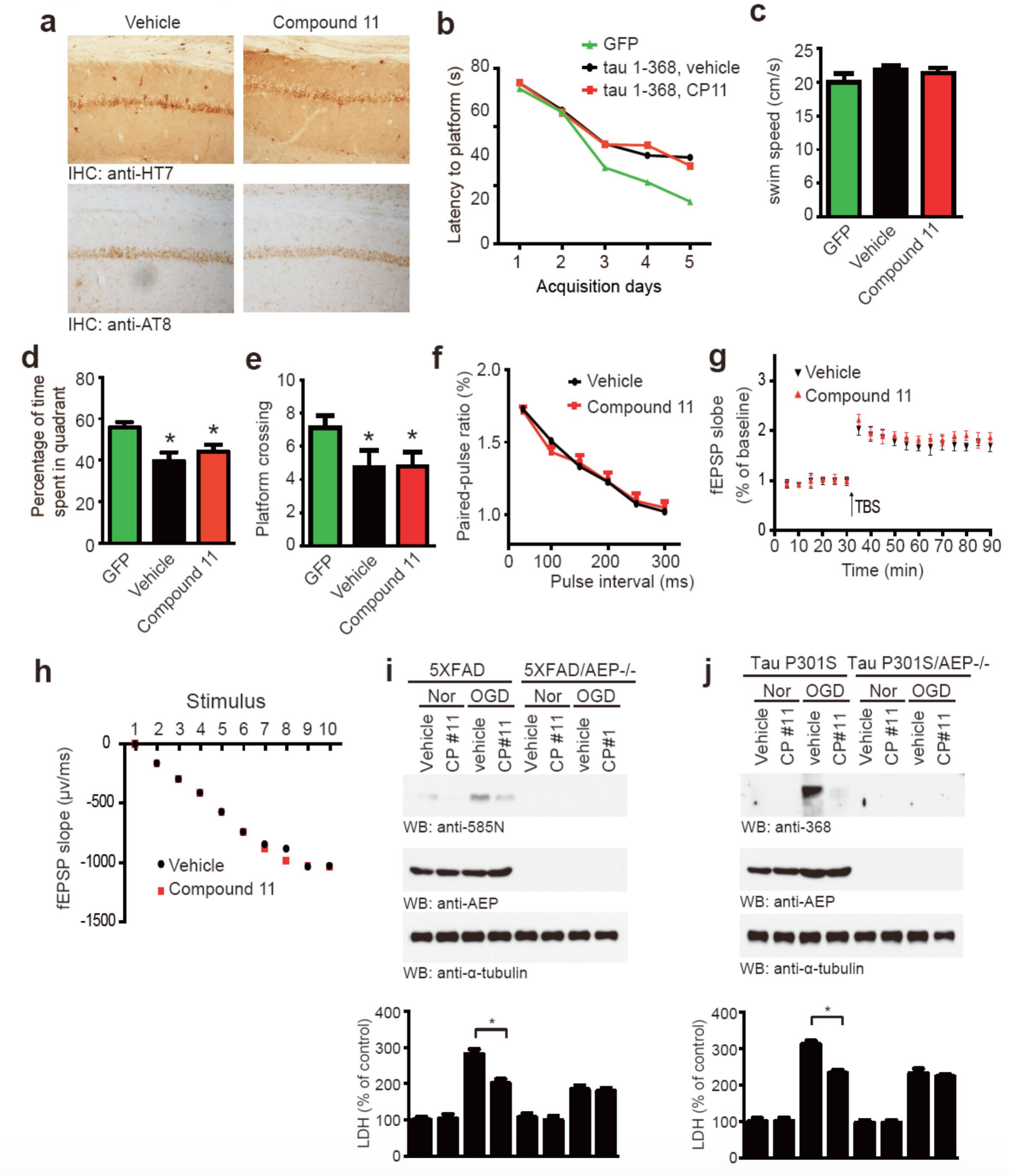

Supplementary Figure 12. Specificity of compound 11 against δ-secretase.

(**a**) Immunohistochemistry showing the expression of human tau and phosphorylated tau in mice injected with AAVs encoding human tau 1-368. Compound 11 did not affect the expression of human tau or the phosphorylated tau.

(**b**) Morris water maze analysis as latency to platform (s) for mice treated with compound 11 or vehicle (mean ± s.e.m.; n = 10 mice per group). Mice overexpressing tau 1-368 show longer time to find the platform compared with mice overexpressing GFP. Compound 11 did not affect the latency to platform.

(**c**) The swim speed of the vehicle- and compound 11-treated mice were similar, indicating similar motor function.

(**d**) The percentage of time spent in the target quadrant in the probe trail was decreased in mice overexpressing tau 1-368 than the GFP-expressing mice, and was not affected by compound 11 treatment. *p < 0.01 compared with GFP group.

(**e**) Number of crossings over the previous location of the target platform was decreased in tau 1-368 mice than GFP-expressing mice, and was not affected by compound 11. *p < 0.01 compared with GFP group.

(**f**) The ratio of paired pulses in tau 1-368 mice was not affect by compound 11 treatment (mean ± s.e.m.; *n* = 3 per group).

(**g**) LTP of fEPSPs was similar between vehicle- and compound 11-treated mice (mean ± s.e.m.; *n* = 6 per group).

(**h**) The input/output (I/O) relation between stimuli intensity and fEPSP slope in vehicle- and compound 11- treated mice. Data are mean ± s.e.m. of 10 mice in each group.

(**i-j**) Effect of compound 11 on OGD-induced APP/tau cleavage and cell toxicity. Compound 11 attenuated OGD-induced APP/tau cleavage and cell toxicity in cultured neurons from 5XFAD mice and tau P301S mice, but not in neurons from 5XFAD/AEP-/- mice and tau P301S/AEP-/- mice. **P* < 0.01.

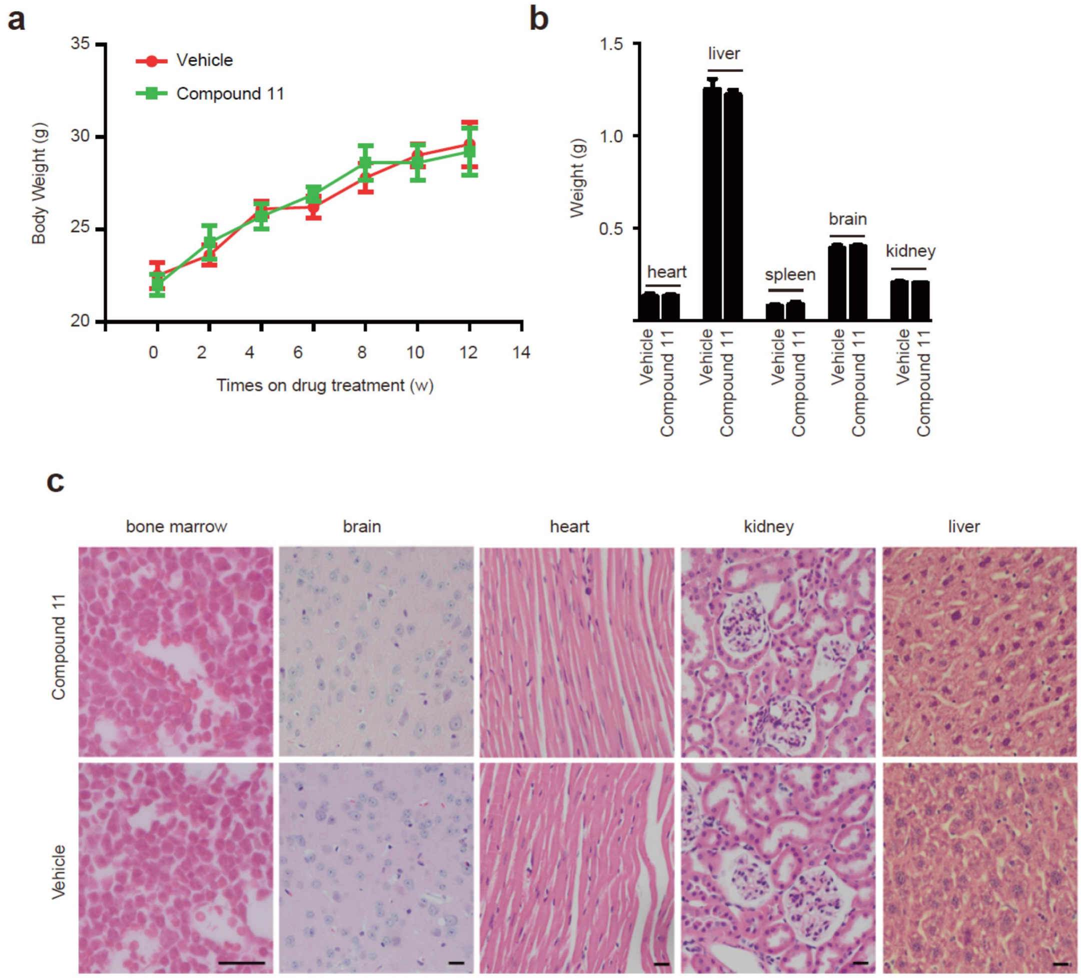

Supplementary Figure 13. Chronic oral administration of compound 11 does not induce toxicity in mice.

(**a**) The body weight of the mice treated with compound 11 or vehicle (mean ± s.e.m.; n = 10 mice per group).

(**b**) The weight of the major organs of the mice treated with compound 11 or vehicle (mean ± s.e.m.; n = 3 mice per group).

(**c**) Hematoxylin and eosin staining of the bone marrow, brain, heart, kidney and liver. Bar=20 µm.

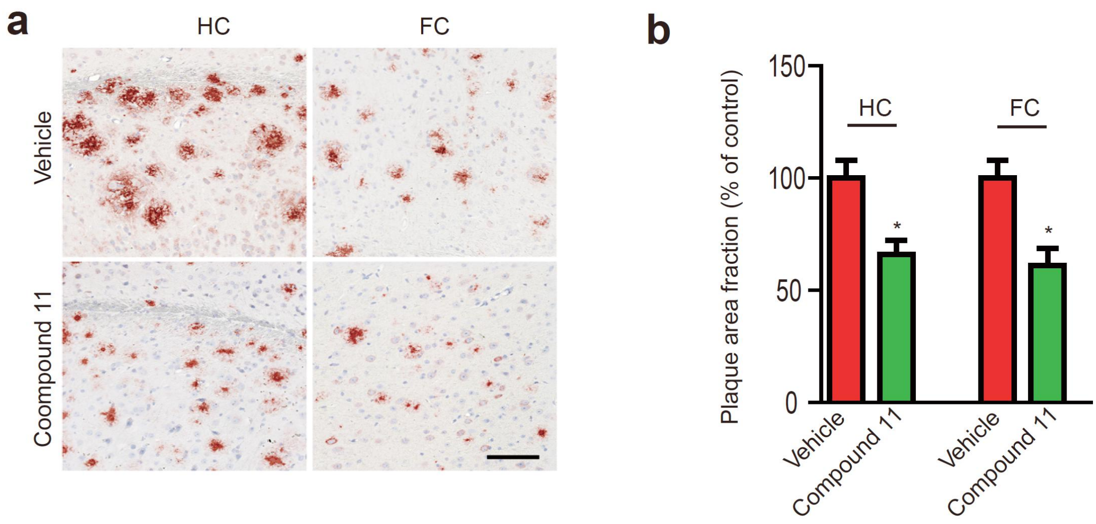

Supplementary Figure 14. Compound 11 reduces amyloid plaque burden in 5XFAD mice.

(**a**) Immunohistochemistry of Aβ deposits in the hippocampus (HC) and frontial cortex (FC) of 5XFAD mice. Scale bar, 100 μm.

(**b**) Quantitative analysis of amyloid plaques. Amyloid deposition in 5XFAD mice was significantly decreased by compound 11. * P <0.01.

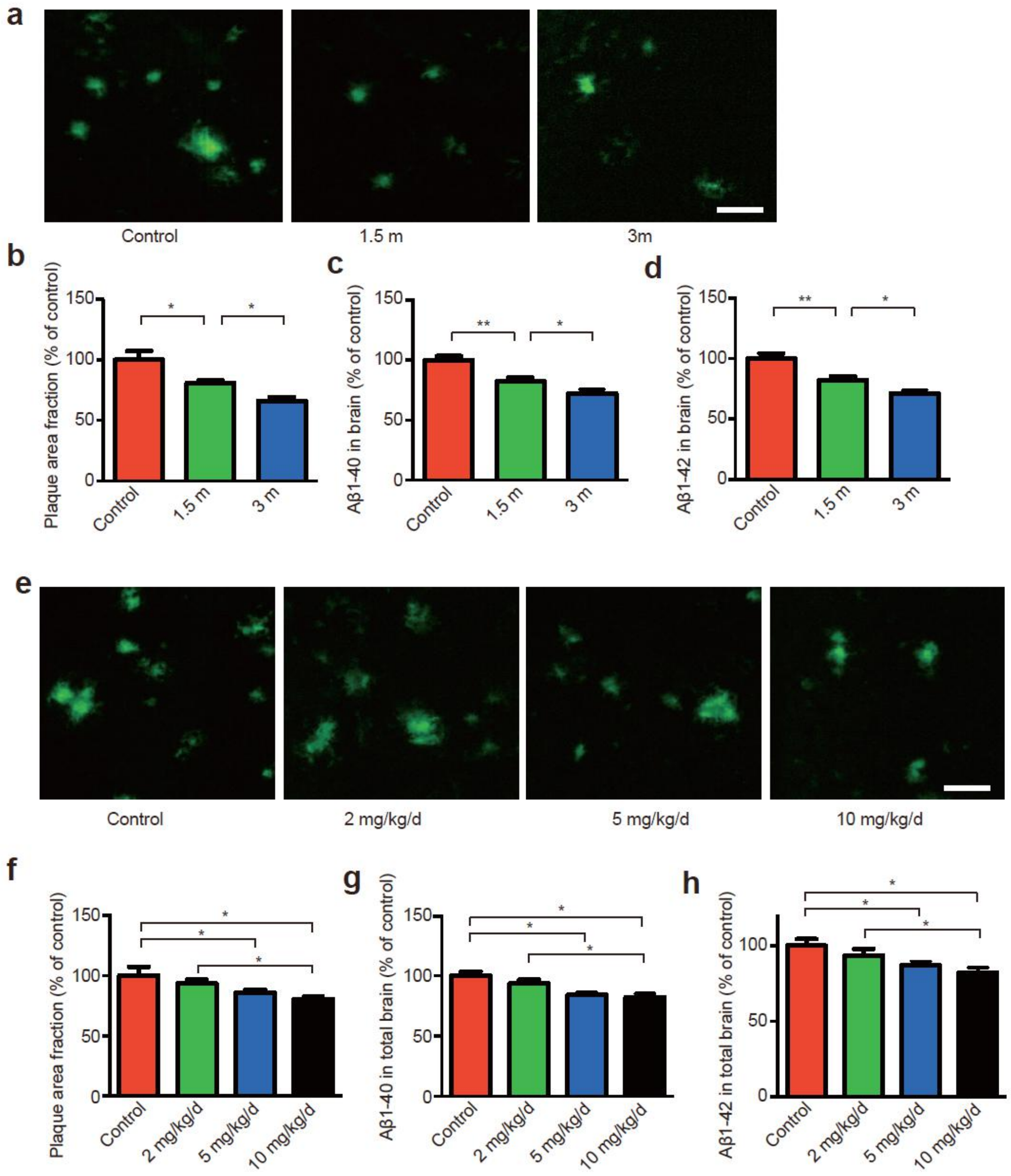

Supplementary Figure 15. Compound 11 time- and dose-dependently attenuates Aβ deposition in 5XFAD mice.

(**a**) Time-dependent effect of compound 11 on the deposition of Aβ in the brain. 5XFAD mice were treated with compound 11 at a dose of 10 mg $kg^{-1}d^{-1}$ for 1.5 month or 3 month, respectively. The deposition of Aβ was determined using thioflavin-S staining. Scale bar, 100 μm.

(**b**) Quantitative analysis of amyloid plaques (mean ± s.e.m.; $*P<0.05$, one-way ANOVA).

(**c-d**) Concentration of Aβ 1-40 and Aβ 1-42 in the brain determined using ELISA (mean ±s.e.m.; $*P<0.05$, one-way ANOVA).

(**e**) Dose-dependent effect of compound 11 on the deposition of Aβ in the brain. 5XFAD mice were treated with compound 11 at a dose of 2, 5, or 10 mg $kg^{-1}d^{-1}$ for 1.5 month. The deposition of Aβ was determined using thioflavin-S staining. Scale bar, 100 µm.

(**f**) Quantitative analysis of amyloid plaques (mean ± s.e.m.; $^*P < 0.05$, one-way ANOVA).

(**g-h**) Concentrations of Aβ 1-40 and Aβ 1-42 in the brain determined using ELISA (mean ± s.e.m.; $^*P < 0.05$, one-way ANOVA).

Figure 3e

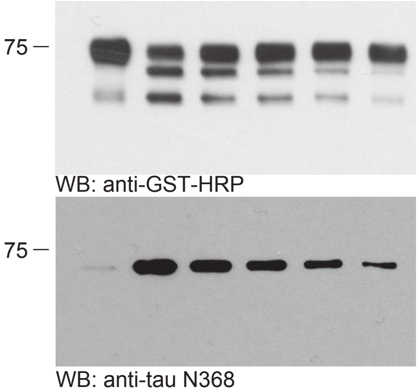

Figure 3f

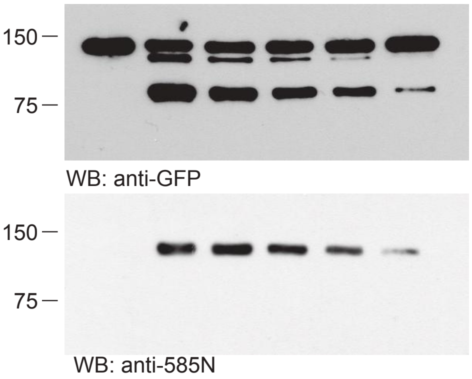

Figure 3h

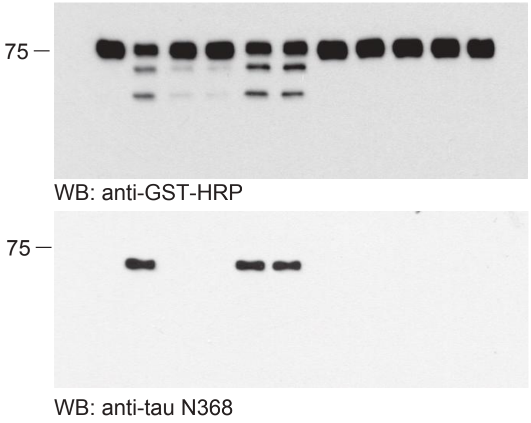

Figure 3g

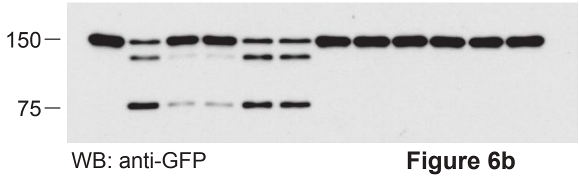

Figure 3i

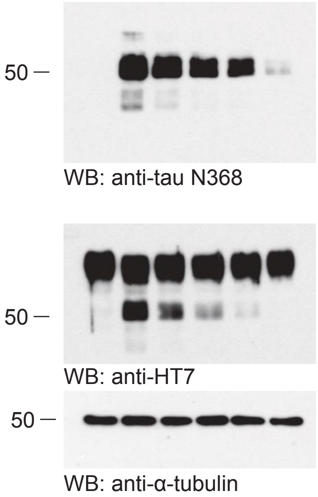

Figure 6b

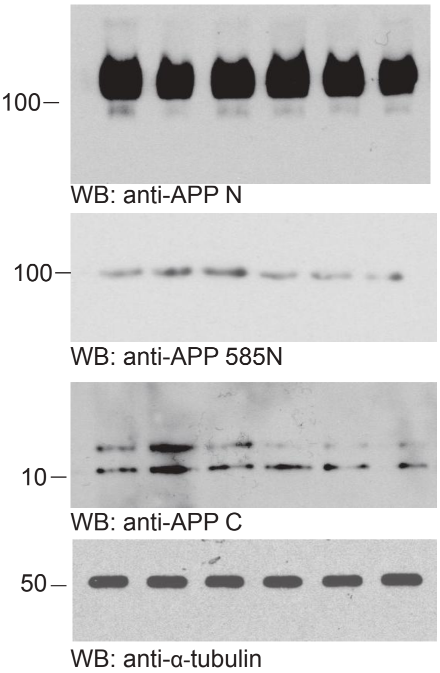

Figure 4b

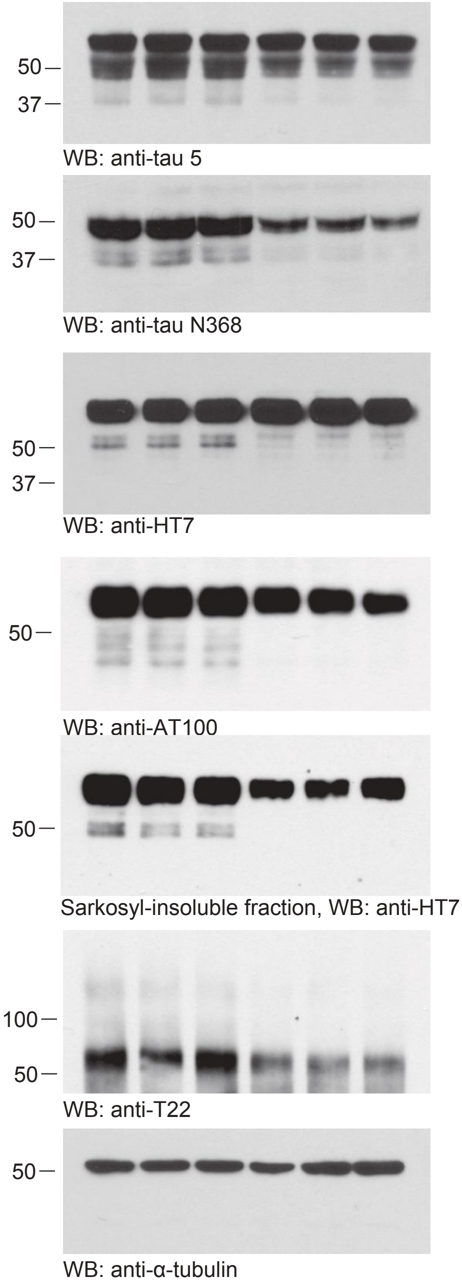

Supplementart Figure 9i

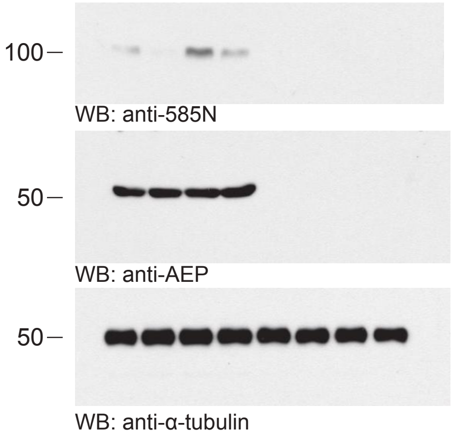

Supplementart Figure 9j

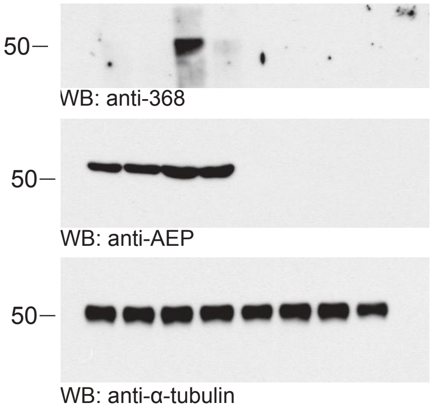

SupplementaryFigure 16. Original scans of Western blots

Supplementary Table 1: X-ray data collection and refinement statistics.

	AEP-Dcmk +compound 11b	AEP-Dcmk +compound 11	apo-AEP +compound 11	apo-AEP +compound 11b
**Data collection**				
Space group	$P4_2$	$P4_2$	$P2_12_12_1$	$P2_12_12_1$
Cell dimensions				
a, b, c (Å)	64.3, 64.3, 78.9	64.3, 64.3, 79.3	43.5, 75,0, 173	43.3, 75.1, 171.9
α, β, γ (°)	90, 90, 90	90, 90, 90	90, 90, 90	90, 90, 90
Resolution (Å)*	49.8–1.9(2.0–1.9)	45.53–2.2(2.3–2.2)	36.79–2.10(2.17–2.1)	36.73–2.0(2.1–2.0)
R_{merge}	0.08(0.55)	0.12(0.71)	0.12(0.82)	0.12(0.54)
$I/\sigma I$	10.5(3.0)	10.3(3.3)	10.2(2.8)	6.5(2.3)
Completeness (%)	99.8(99.9)	96.4(100)	99.9(99.8)	95.8(96.5)
Redundancy	5.0(5.0)	10.0(9.3)	6.0(5.7)	3.7(3.4)
**Refinement**				
Resolution (Å)	49.9–1.95	45.5–2.20	36.8–2.10	36.7–2.00
No. reflections	22169	13753	32251	35022
R_{work} / R_{free}	21.8/25.0	24.4/27.3	22.1/25.3	22.2/25.1
No. atoms				
Protein	2139	2139	4192	4192
Ligand/ion	71	65	142	145
Water	84	70	254	272
Overall B-factors (Å^2)	32.6	39.4	28.3	27.4
R.m.s deviations				
Bond lengths (Å)	0.005	0.005	0.005	0.005
Bond angles (º)	1.09	1.06	1.03	1.02

The structures were determined from single crystals.

*Highest resolution shells are shown in parentheses.

Supplementary Table 2. Small molecule screening data

Category	Parameter	Description
Assay	Type of assay	Biochemical
	Target	AEP
	Primary measurement	Detection of the generation of AMC fluorescence intensity at Ex: 340 nm and Em: 480 nm for AEP enzyme activity
	Key reagents	AEP+/+ mouse kidney lysate and Z-Ala-Ala-Asn-AMC
	Assay protocol	Please see method
	Additional comments	The assay was carried out in kinetic mode
Library	Library size	54,384 compounds
	Library composition	Chemical diversity libraries
	Source	Asinex Corporation; ChemDiv
	Additional comments	
Screen	Format	1536-well plates
	Concentration(s) tested	16.7 μM
	Plate controls	S/B > 4
	Reagent/ compound dispensing system	MultiDrop Combi (Thermo-Fisher); Beckman NX
	Detection instrument and software	Envision multilabel plate reader; Envision Manager
	Assay validation/QC	Z' > 0.5
	Correction factors	
	Normalization	
	Additional comments	Screening was performed by Emory Chemical Biology Discovery Center (http://www.pharm.emory.edu/ECBDC/)
Post-HTS analysis	Hit criteria	% of Control < 50; excluding fluorescence interference compounds
	Hit rate	1.5% (749 hits)
	Additional assay(s)	Primary screening assay using AEP (+/+) Kidney lysate; Counter screening assay using AEP (-/-) kidney lysate
	Confirmation of hit purity and structure	Re-purchased and confirmed hits
	Additional comments	

Supplementary Table 3: Caco-2 permeability and BBB-PAMPA permeability

Compound	A ->B P_{app} (10^{-6} cm•s^{-1})	B -> A P_{app} (10^{-6} cm•s^{-1})	R_E	P_e (10^{-6} cm•s^{-1})
Ranitidine	0.8	2.5	3.2	NA
Warfarin	28.5	12.6	0.4	NA
Talinolol	0.3	6.0	23.9	NA
Theophylline	NA	NA	NA	0.12
Verapamil	NA	NA	NA	17.2
11	35.0	7.0	0.2	> 25
12	1.4	3.7	2.7	< LLOQ
31	19.3	17.4	0.9	< LLOQ
38	1.1	20.3	18.2	0.007
64	< LLOQ	< LLOQ	NA	ND

For CaCo-2 permeability assay, cells were incubated with 10 µM of these reagents in buffers for 2 h, and the receiver side buffer is removed for analysis by LC/MS/MS. Efflux ratio (R_E) > 2 indicates a significant efflux activity, an indication of potential substrate for PGP or other active transporters. For BBB-PAMPA permeability assay, the filter membrane was coated with 4 µl of a 20 mg ml^{-1} porcine brain lipid in dodecane. 200 µl of the compound solution was added to the donor well. The acceptor well was filled with 200 µl of transport buffer. The acceptor filter plate was carefully placed on to the donor plate to create a sandwich. The plate was left undisturbed for 18 h. Samples of the donor and acceptor wells were analyzed by LC/MS/MS and the effective permeability (Pe) was calculated. LLOQ: Lower Limit of Quantification; P_{app}: apparent permeability. ND, Peak not detected due to bioanalysis issue. NA, not tested.

Supplementary Table 4: Liver microsomal stability

Compound	Species	Mean Remaining Parent (with NADPH)	Mean Remaining Parent (NADPH-free)
Verapamil	Human	4.6%	101%
	Mouse	2.6%	101%
Warfarin	Human	96%	104%
	Mouse	91%	100%
11	Human	76%	93%
	Mouse	20%	103%
12	Human	1%	1%
	Mouse	1%	0%
31	Human	12%	75%
	Mouse	16%	86%
38	Human	88%	107%
	Mouse	98%	99%
64	Human	ND*	ND*
	Mouse	ND*	ND*

The test agents were incubated in duplicate with microsomes at 37 ºC for 30 min. The reaction contained microsomal protein in 100 mM potassium phosphate, 2 mM NADPH, 3 mM $MgCl_2$, pH 7.4. A control was run for each test agent omitting NADPH to detect NADPH-free degradation. Data are reported as % remaining of parent compound. ND, Peak not detected due to bioanalysis issue (poor ionization).

Supplementary Table 5: CYP inhibition

Compound	Test Concentration (μM)	CYP3A4 -Midazolam	CYP3A -Testosterone	CYP2C9	CYP2D6	CYP2C19	CYP1A2
11	10	8.0%	21.6%	5.6%	21.9%	6.8%	37.0%
	3	3.6%	4.1%	4.3%	7.8%	4.4%	13.1%
12	10	-2.5%	27.6%	-2.7%	-2.6%	29.4%	44.5%
	3	-2.5%	14.2%	4.3%	2.9%	13.4%	28.4%
31	10	18.1%	0.7%	69.2%	1.4%	55.7%	10.3%
	3	-0.9%	2.5%	47.8%	9.9%	37.7%	13.5%
38	10	10.4%	2.4%	17.1%	0.9%	9.5%	10.5%
	3	7.3%	-6.0%	15.6%	-5.4%	-12.0%	-7.6%
64	10	10.1%	-0.1%	7.9%	58.3%	20.4%	22.2%
	3	0.6%	1.5%	4.6%	34.6%	31.6%	23.7%

Test agent was incubated (three wells per condition) with microsomes at 37 °C. Control incubations containing vehicle or reference inhibitors were run along side the test agents. The final assay contained test agent and probe substrates at the indicated concentration, 2 mM NADPH, 3 mM $MgCl_2$ in 50 mM potassium phosphate buffer, pH 7.4. The final microsomal concentration was 0.5 mg ml^{-1}. At the end of 10 min incubation, the assay was stopped by the addition of acetonitrile serving as internal standard, the samples were centrifuged, and the amount of probe metabolite in the supernatant was determined by LC/MS/MS.

Suppplementary Table 6. Assigned chemical shifts of compound 11 recorded in DMSO d6 at 298 K referenced to Tetramethylsilane (TMS).

**Label**	**^{1}H data**<br>**δ [ppm] / J [Hz]**	**^{13}C data**<br>**δ [ppm]**
1′	3.17	50.2
2′	3.78	66.0
5	6.49 / 7.7	106.2
6	6.25 / 7.7	115.2
NH_2	5.78	

Supplementary Table 7. Assigned chemical shifts of compound 11b recorded in DMSO d6 at 298 K referenced to Tetramethylsilane (TMS).

**Label**	**^{1}H data**<br>**δ [ppm] / J [Hz]**	**^{13}C data**<br>**δ [ppm]**
1′	2.90	50.4
2′	3.70	66.2
1″	2.79	50.6
2″	3.72	66.4
2	6.55 / 2	108.4
5	6.59 / 8.5	114.8
6	6.46 / 8.5 / 2	111.9

Supplementary Table 8: Pharmacokinetics of intravenously (IV) and orally administered compound 11

IV compound 11 single dose

• •	Time (hours)	Animal No. 101M	102M	103M	Mean	std. Dev.
	0.08	240.4	232.5	216.9	229.9	12.0
	0.25	16.5	55.3	42.6	38.1	19.8
	0.50	6.4	11.1	22.3	13.3	8.2
	1.00	2.1	3.4	4.9	3.5	1.4
	2.00	4.1	BLQ	3.4	3.8	0.5
	4.00	BLQ	3.1	BLQ	3.1	NA
	8.00	BLQ	BLQ	BLQ	NA	NA
• •	24.00	BLQ	BLQ	BLQ	NA	NA
$AUC_{(0-t)}$	ng/mL*h	51.77	71.49	60.21	61.15	9.89
$AUC_{(0-\infty)}$	ng/mL*h	51.82	71.49	60.30	61.20	9.86
$MRT_{(0-t)}$	h	0.20	0.45	0.26	0.30	0.13
V_Z	L/kg	14.43	6.45	11.42	10.77	4.03
CL_Z	L/h/kg	38.59	27.98	33.17	33.25	5.31
$T_{1/2Z}$	h	0.26	0.16	0.24	0.22	0.05
C_{max}	ng/mL	240.4	232.5	216.9	229.9	12.0

Oral compound 11 single dose

• •	Time Hours	Animal No. 201M	202M	203M	Mean	std. Dev.
	0.25	199.4	819.9	321.1	446.8	328.8
	0.50	77.8	48.7	116.9	81.1	34.2
	1.00	33.9	12.2	35.4	27.2	13.0
	2.00	11.3	6.9	21.8	13.3	7.7
	4.00	16.7	6.7	11.3	11.6	5.0
	8.00	BLQ	3.4	4.2	3.8	0.6
• •	24.00	BLQ	BLQ	BLQ	NA	NA
$AUC_{(0-t)}$	ng/mL*h	138.1	269.6	225.7	211.1	67.0
$AUC_{(0-\infty)}$	ng/mL*h	138.8	288.1	240.8	222.6	76.3
$MRT_{(0-t)}$	h	1.11	0.84	1.49	1.15	0.33
V_Z/F	L/kg	57.2	190.9	153.4	133.9	69.0
CL_Z/F	L/h/kg	72.05	34.71	41.52	49.43	19.88
$T_{1/2Z}$	h	0.55	3.81	2.56	2.31	1.65
T_{max}	h	0.25	0.25	0.25	0.25	0.00
C_{max}	ng/mL	199.4	819.9	321.1	446.8	328.8
F	%	45.16	88.18	73.80	69.05	21.90

Oral compound 11 10 mg $kg^{-1}d^{-1}$, 3 months, 1 hour after last dose

Sample	Compound #11 in the serum (ng ml^{-1})	Concerntation in the brain (ng g^{-1})
Sample #1	184	206
Sample #2	59	87

Supplementary Table 9: Complete blood count (CBC), liver function test and renal function test of mice treated with vehicle or compound 11

Parameter	Vehicle	Compound 11
White blood cell ($X10^9\ l^{-1}$)	1.81 ± 0.48	1.37 ± 0.08
Red blood cell ($X10^{12}\ l^{-1}$)	8.15 ± 0.44	7.38 ± 1.15
Hemoglobin (g dl^{-1})	13.8 ± 1.14	12.7 ± 0.94
MCH (pg)	16.97 ± 0.85	17.53 ± 3.42
MCHC (%)	36.1 ± 1.76	40.8 ± 5.07
platelet ($X10^9\ l^{-1}$)	433.67 ± 233.9	491.26 ± 69.8
ALB (g dl^{-1})	3.33 ± 0.74	3.57 ± 0.06
ALP (U L^{-1})	71.00 ± 47.29	35.25 ± 28.48
ALT (U L^{-1})	81.67 ± 55.83	64.25 ± 34.60
TBIL (mg dl^{-1})	0.30 ± 0	0.15 ± 0.17
BUN (mg dl^{-1})	31.00 ± 8.19	26.75 ± 3.30
CA (mg dl^{-1})	9.13 ± 0.35	9.22 ± 0.49

MCH: mean corpuscular hemoglobin; MCHC: mean corpuscular hemoglobin concentration; ALB: albumin; ALP: alkaline phosphatase; ALT: alanine aminotransferase; TBIL: total bilirubin; BUN: blood urea nitrogen; CA: creatinine
